# Pain in Long COVID: A scoping review of clinical characteristics and patterns of manifestation*

**DOI:** 10.1590/1518-8345.7836.4777

**Published:** 2025-12-01

**Authors:** Ana Cristina Ribeiro La Scaléa, Sílvia Carla da Silva André Uehara

**Affiliations:** 1 Universidade Federal de São Carlos, Departamento de Enfermagem, São Carlos, SP, Brazil. Universidade Federal de São Carlos Departamento de Enfermagem SP São Carlos Brazil; 2 Scholarship holder at the Coordenação de Aperfeiçoamento de Pessoal de Nível Superior (CAPES), Brazil. Scholarship holder at the Coordenação de Aperfeiçoamento de Pessoal de Nível Superior Brazil

**Keywords:** COVID-19, Post-Acute COVID-19 Syndrome, Signs and Symptoms, Pain, Chronic Pain, Public Health

## Abstract

to map the available scientific evidence on the clinical characteristics and patterns of pain manifestation (location, frequency, duration, intensity, and quality) in individuals with Long COVID.

a scoping review of publications from March 2020 to June 2024, indexed across four databases. Study selection was conducted by two independent, blinded reviewers. Data were extracted using a standardized instrument and analyzed descriptively.

nineteen studies were included, indicating that pain affects individuals across all age groups, with higher prevalence among women, primarily involving the head, neck, shoulder, lower back, and hip. Pain frequency ranged from daily to monthly episodes, with duration exceeding one year in some cases. Intensity varied from mild to severe, and pain characteristics were diverse, with descriptors including burning, pressure, colicky, and throbbing pain.

the clinical characteristics and patterns of pain manifestation in Long COVID are diverse. However, there is a paucity of studies providing detailed analyses of pain features and the influence of individual variables. These findings should guide future research and clinical practice toward a more comprehensive and contextualized assessment of pain in Long COVID.

## Highlights

**(1)** Regions most frequently affected by pain: chest, head, neck, shoulder, lower back, and hip. **(2)** Intensity ranges from mild to severe. **(3)** Duration varies from a few months to several years. **(4)** Exacerbation of preexisting pain following viral infection.

## Introduction

Coronavirus disease (COVID-19) has caused more than 280 million cases and 5 million deaths worldwide^(1)^. In May 2023, the World Health Organization (WHO) declared the end of the public health emergency of international concern imposed by COVID-19; however, the impact on the health conditions of infected individuals extends beyond survival from severe forms of the disease^(1-2)^.

Following the acute viral infection, reports of post-acute sequelae of COVID-19—also referred to as Long COVID—began to emerge. This condition is characterized by the development or persistence of symptoms lasting for at least two months after 12 weeks from the acute infection^(3)^. In this context, the incidence of Long COVID has been reported to range from 10% to 70% of acute cases and can occur in individuals who experienced both severe and mild forms of the disease^(4-5)^.

Although the pathogenic mechanism of Long COVID is not yet fully elucidated, scientific evidence has identified more than two hundred symptoms, highlighting the multisystemic nature of the condition, in which some symptoms may last for weeks or months, and others for an indefinite period^(3-4)^.

Among the listed symptoms of Long COVID, pain stands out, defined as “an unpleasant sensory and emotional experience associated with actual or potential tissue damage”^(3-4,6)^. Acute pain serves as a protective mechanism, signaling the presence of a potentially harmful stimulus; however, any pain persisting beyond the normal tissue healing time—approximately three months—is classified as chronic pain^(7-8)^.

It is important to note that chronic pain affects approximately 30% of individuals worldwide^(9)^ and pain as a symptom of Long COVID is expected to further increase this rate, given the lack of unified guidelines for managing patients with Long COVID. In this context, assessing pain characteristics—such as location, intensity, frequency, and quality—is essential to determine whether it is a symptom of Long COVID or attributable to other underlying conditions^(5)^.

Additionally, health care professionals must consider factors such as sex, age, presence of comorbidities, and hospitalization history due to infection with Severe Acute Respiratory Syndrome Coronavirus 2 (SARS-CoV-2), to help identify patients at risk of developing Long COVID^(10)^. A careful evaluation of these aspects enables a more targeted and personalized approach, contributing to more accurate diagnosis and effective management of the signs and symptoms associated with Long COVID^(5)^.

In this context, nursing plays a key role in the assessment, monitoring, and implementation of effective interventions for individuals with Long COVID, particularly in pain management. Accordingly, the importance of nursing diagnosis is underscored, as it guides evidence-based and individualized clinical decision-making. The role of nurses, grounded in the nursing care process, is fundamental for the early identification of symptoms, care planning, and the promotion of physical and emotional comfort, thereby contributing to comprehensive patient rehabilitation^(11-14)^.

In the context of Long COVID, pain can affect multiple aspects of individuals’ lives, including their quality of life and social well-being. Furthermore, individuals with chronic pain tend to have higher rates of work absenteeism and make more frequent use of health care resources compared to those without chronic pain^(15)^.

In this scenario, despite the significant increase in studies focusing on Long COVID manifestations, the emphasis has remained on respiratory, neurological, and psychological symptoms^(3-5)^, while pain is often treated as a secondary manifestation, without detailed analysis of its clinical characteristics. Thus, there is a clear gap in the understanding of pain characteristics in patients with Long COVID, which hinders the development of targeted, evidence-based therapeutic strategies.

Understanding the clinical characteristics and patterns of pain in Long COVID may help distinguish post-COVID pain from other painful conditions, as well as strengthen professional practice by supporting more well-founded clinical decision-making, fostering the training of health care teams in the early recognition of this manifestation, and contributing to the planning of interdisciplinary actions that address the specific needs of this population^(3-5,10-14)^. Therefore, this study aims to map the available scientific evidence on the clinical characteristics and patterns of pain manifestation (location, frequency, duration, intensity, and quality) in individuals with long-duration coronavirus disease (Long COVID).

## Method

### Study design

This study is a scoping review, structured according to the Joanna Briggs Institute (JBI) manual for evidence synthesis, which comprises the following steps: identification of the research question, identification of relevant studies, study selection, data extraction, charting, summarization and reporting of results, and dissemination of findings^(16)^. The protocol for this study has been published in the Open Science Framework DOI 10.17605/OSF.IO/YVXDF.

### Identification of the research question

To construct the research question, we used a strategy recommended for scoping reviews that helps identify key topics—the PCC framework, a mnemonic that stands for P (Population: individuals with pain as a symptom of Long COVID), C (Concept: pain), and C (Context: Long COVID). Accordingly, the research question was defined as follows: What are the clinical characteristics and patterns of pain manifestation (location, frequency, duration, intensity, and quality) in patients with Long COVID?

### Search strategy

Different strategies were adopted to identify scientific articles in electronic databases, namely: PubMed Central, Web of Science, Scopus, and the Virtual Health Library, along with manual searches of the reference lists of selected articles. Database access was carried out through the CAPES Journal Portal, using identification via the Federated Academic Community (CAFe), as a means of standardizing data collection across databases.

Searches were conducted using descriptors and/or alternative terms in English, drawn from the Health Sciences Descriptors (DeCS) and Medical Subject Headings (MeSH), as presented in Figure 1

**Figure 1-:** Search strategy across electronic databases. São Carlos, SP, Brazil, 2024

**Electronic database**	**Search strategy**
PubMed Central	(Long COVID[Abstract] OR Post-Acute COVID-19 Syndrome[Abstract]) AND Pain[Abstract]
Web of Science	((AB=(“Long COVID”)) OR AB=(“Post-Acute COVID-19 Syndrome”)) AND AB=(Pain)
Virtual Health Library	(ab:(long COVID)) OR (ab:(post-acute COVID-19 syndrome)) AND (ab:(pain)) AND instance:“lilacsplus”
Scopus	(ABS(“Long COVID”) OR ABS(“Post-Acute COVID-19 Syndrome”) AND ABS(pain)) AND PUBYEAR > 2019 AND PUBYEAR < 2025 AND (LIMIT-TO(DOCTYPE,“ar”))

### Eligibility criteria

The inclusion criteria comprised primary studies published in Portuguese, English, and Spanish between March 2020 and June 2024. Excluded were duplicate articles, opinion papers, editorials, reviews, website content, studies whose titles and abstracts were not aligned with the research objective, and articles not available in full text and free of charge.

### Data extraction, treatment, and analysis

The article search was conducted in electronic databases in July 2024, and the study selection process took place from August to October 2024. To support the selection process, after implementing the search strategy in each database, the references were imported into the StArt (State of the Art through Systematic Review) web application^(17)^, a review tool developed by the Software Engineering Research Laboratory (LaPES) at the Federal University of São Carlos (UFSCar). Study selection occurred in two stages: initial screening of titles and abstracts, followed by full-text review. Eligible studies were retrieved in full and assessed by two researchers. In both phases, discrepancies were discussed until consensus was reached, followed by the final selection.

Data extraction was carried out using a spreadsheet instrument developed in Microsoft Excel, with fields designed to capture relevant information from the primary studies, including study identification (authors, year, country of publication, and objective), methodological characteristics (study design and sample size), and main findings related to demographic and clinical characteristics, as well as pain-related features reported as symptoms of Long COVID (location, frequency, duration, intensity, and quality). This instrument was developed to ensure uniformity and to facilitate the organization of data, while also allowing for subsequent quantitative and qualitative analyses of the results.

The study selection process is shown in a flow diagram, and the findings extracted from the publications are displayed in figures (charts) in descriptive format.

The conduct of this review also followed the recommendations of the Preferred Reporting Items for Systematic Reviews and Meta-Analyses Extension for Scoping Reviews (PRISMA-ScR)^(18)^.

### Ethical considerations

Regarding the ethical aspects of the research, no discrimination was applied in the selection of articles or studies, and the criterion of individual blinding was maintained.

## Results

**Figure 2-:**
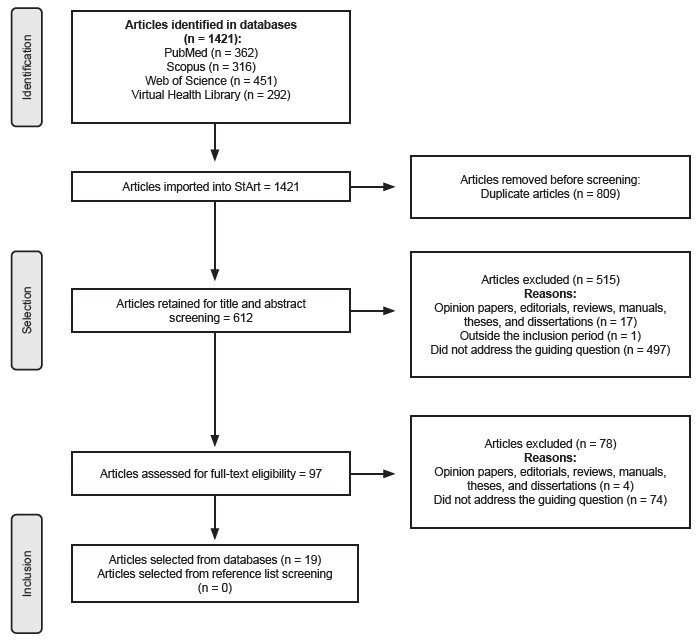
Reference flowchart: inclusion and exclusion of articles. São Carlos, SP, Brazil, 2024

A total of 1,421 articles were identified across the databases. Of these, 809 were excluded as duplicates, 515 after screening titles and abstracts, and 78 after full-text review. Consequently, 19 articles addressing pain as a symptom of Long COVID were included in the study (Figure 2).

All 19 (100%) publications included in this scoping review were published in English. Regarding the countries where the studies were conducted, 5 (26.3%) were carried out in the United States, 4 (21%) in Turkey, 3 (15.6%) in Spain, and 1 (5.3%) in each of the following countries: Iran, Indonesia, Cyprus, Japan, Brazil, Mexico, and the United Kingdom.

With respect to study design, 9 (47.3%) were cross-sectional studies, 5 (26.3%) cohort studies, 4 (21.1%) case studies, and 1 (5.3%) case-control study. Relevant information from each selected article—including authors, year of publication, country where the study was conducted, study design and sample, and main results—is presented in Figure 3.

**Figure 3-:** Description of the articles, according to author, year, country, objective, study design, and demographic and clinical characteristics. São Carlos, SP, Brazil, 2024

**N** *	**Author, year, and country**	**Objective**	**Study design**	**Demographic and clinical characteristics**
1	Arostegui, 2021, United States ^( 19 )^	To describe a case report of gastrointestinal symptoms in a child three months after COVID-19 ^†^ diagnosis.	Case study.	N ^‡^ = 1, 11 years old, female. The child presented gastrointestinal symptoms during the acute phase of COVID-19 ^†^ infection, which persisted after three months. After three weeks of therapy, abdominal pain remained unresolved.
2	Savarraj, 2021, United States ^( 20 )^	To characterize long-term neurological outcomes after COVID-19 ^†^ .	Cohort study.	N ^‡^ = 58, mean age 49.2 years, 54% male. Included individuals with a history of hospitalization for COVID-19 ^†^ . The most common symptom in the sample was pain (60%). Those who reported pain were significantly younger (45 vs. 53 years; P ^§^ < 0.05). Women presented a higher frequency of pain, although the difference was not statistically significant, and there was no variation regarding ethnicity or prior medical history.
3	Azadvari, 2022, Iran ^( 21 )^	To assess the prevalence of different musculoskeletal symptoms and their associated factors in patients with Long COVID ^†^ .	Cross-sectional study.	N ^‡^ = 239, mean age 37.96 years; 69.9% female. In the sample, myalgia (80.3%) and headache (62.3%) were the most common symptoms. Body mass index and days lost due to COVID-19 ^†^ were significantly higher among patients who experienced chest pain, leg pain, arthralgia, and myalgia for longer period (P ^§^ < 0.05). Female sex was associated with a higher risk of being bedridden due to pain (P ^§^ = 0.037).
4	Peñas, 2022, Spain ^( 22 )^	To identify the correlation between pain extent and sensory symptoms associated with sensitization, as well as cognitive and psychological variables, in COVID-19 ^†^ survivors with post-COVID ^†^ pain.	Cross-sectional study.	N ^‡^ = 146, mean age 57.5 years; 54.11% female. Included individuals with a history of hospitalization for COVID-19 ^†^ . The greater the pain extent, the lower the pain intensity (P ^§^ = 0.014). The association between pain extent and pain intensity was significant, although still weak, in men (P ^§^ = 0.006), but not in women with post-COVID ^†^ pain.
5	Peñas, 2022, Spain ^( 23 )^	To investigate the association between serological biomarkers of COVID-19 ^†^ severity at hospital admission and post-COVID ^†^ pain symptoms in previously hospitalized COVID-19 ^†^ survivors.	Cohort study.	N ^‡^ = 412, mean age 62 years old; 53.9% male. The prevalence of pain was 42.7% and 36.2% at 6.8 and 13.2 months after hospital discharge, respectively. Patients who developed post-COVID ^†^ pain six months after discharge presented a higher number of symptoms at hospital admission and a greater number of comorbidities compared to those without post-COVID ^†^ pain at six months. Among those who developed post-COVID ^†^ , 51.7% already had preexisting pain, with pain exacerbation in 16.4% of cases.
6	Güven, 2022, Turkey ^( 24 )^	To identify predictors of Long COVID ^†^ by analyzing clinical, laboratory, and demographic characteristics of children with COVID-19 ^†^ .	Cohort study.	N ^‡^ = 500, mean age 12.75 years (COVID-19 ^†^ positive) and 12.50 years (control group). In both groups, 50.2% were female. In total, 12.4% of children reported Long COVID ^†^ symptoms. Symptoms were significantly more common in patients who presented signs of upper respiratory tract infection at physical examination (P ^§^ = 0.025).
7	Mutiawati, 2022, Indonesia ^( 25 )^	To determine the characteristics of headache in post-COVID-19 ^†^ patients in Indonesia.	Cross-sectional study.	N ^‡^ = 215, 45.6% aged between 30 and 39 years; 69.8% female. A total of 72.1% of participants experienced headache during hospitalization or self-isolation for COVID-19 ^†^ . Factors such as physical activity, noise, and bright light, or a combination of these, aggravated headache, while rest, analgesics, and a combination of rest and analgesics helped relieve pain.
8	McHarg, 2022, United States ^( 26 )^	To systematically characterize ocular symptoms found in most non-hospitalized individuals with COVID-19 ^†^ infection.	Cross-sectional study.	N ^‡^ = 229, mean age 42.5 years; 78.1% female. Eye pain was reported in 24.5% of the sample. Ocular symptoms were more frequent in individuals with previous ocular history; only 13.3% sought ophthalmologic care, and among these, 40.9% had their symptoms attributed to COVID-19 ^†^ .
9	Topal, 2022, Turkey ^( 27 )^	To investigate prolonged musculoskeletal pain as a symptom of post-COVID-19 ^†^ condition.	Cross-sectional study.	N ^‡^ = 501, mean age 39 years; 51.3% male. Sixty-nine participants reported prolonged post-COVID ^†^ pain. Among them, 58% were women, with no significant sex differences. The incidence of overall comorbidity or the presence of one or more additional conditions concomitant with COVID-19 ^†^ was higher in patients with prolonged pain.
10	Zha, 2022, United States ^( 28 )^	To describe a case report of myofascial pain after COVID-19 ^†^ infection.	Case study.	N ^‡^ = 1, 59 years old, male. The patient reported inability to perform activities of daily living due to persistent exertional dyspnea, brain fog, and diffuse myalgia. After 12 months, symptoms worsened, associated with a significant increase in psychosocial stress, which contributed to their exacerbation.
11	Zis, 2022, Cyprus ^( 29 )^	To estimate the prevalence of chronic pain in general and neuropathic pain in particular, to describe the most common pain syndromes, and to identify pain determinants in a random population of COVID-19 ^†^ survivors.	Cross-sectional study.	N ^‡^ = 90, mean age 47.5 years; 62.2% female. A total of 63.3% of participants reported chronic pain, which was more frequent among older individuals (50 vs. 42 years) and women. After adjustment for age and sex, headache during COVID-19 ^†^ was a significant predictor of neuropathic pain, with a 4.9-fold higher risk.
12	Mateo, 2023, Spain ^( 30 )^	To assess health-related quality of life and pain characteristics in patients with Long COVID ^†^ and to compare pain location with those who successfully recovered from COVID-19 ^†^ .	Case-control study.	N ^‡^ = 213, in the Long COVID ^†^ group, mean age 44.99 years; 75.26% female. The prevalence, intensity, and generalization of pain were significantly higher in individuals with Long COVID ^†^ compared to recovered individuals and healthy controls.
13	Erden, 2023, Turkey ^( 31 )^	To investigate musculoskeletal symptoms, type of pain, and the effect on quality of life in patients presenting with pain after COVID-19 ^†^ .	Cross-sectional study.	N ^‡^ = 97, mean age 46.5 years old; 69.1% male. The mean visual analog scale score among women, as well as the number of female patients who developed myalgia after COVID-19 ^†^ were significantly higher than those of men (P ^§^ < 0.05). Individuals with preexisting musculoskeletal pain experienced exacerbation of their pain after COVID-19 ^†^ infection.
14	Fujita, 2023, Japan ^( 32 )^	To clarify the characteristics of headaches in patients with Long COVID ^†^ focusing on patient history and clinical conditions, and to assess the impact of headaches on these patients’ quality of life.	Cohort study.	N ^‡^ = 482, mean age 37 years; 56% female. A total of 23.4% of participants reported headache. In the acute phase of COVID-19 ^†^ , most cases of Long COVID ^†^ were mild in both the headache group (88.5%) and the non-headache group (82.7%).
15	Rodrigues, 2023, Brazil ^( 33 )^	To evaluate the clinical characteristics of headache in Long COVID ^†^ .	Cross-sectional study.	N ^‡^ = 102, 62.7% over 39 years old; 81.37% female. Headache in Long COVID ^†^ was more frequently observed in women over 39 years old. A total of 49.0% of participants had a previous history of headache and reported worsening of pain during the prolonged COVID ^†^ phase.
16	Yadav, 2023, United States ^( 34 )^	Case reports of five patients who developed joint pain several weeks after recovery from acute COVID-19 ^†^ infection.	Case study.	N ^‡^ = 5, female, aged between 19 and 61 years. Three cases reported in the study presented pain symptoms less than one month after COVID-19 ^†^ therefore, they were not considered Long COVID ^†^ . First case: 45-year-old woman with morbid obesity and osteoarthritis, who showed 70–80% improvement of symptoms after 3 months of corticosteroid therapy. Second case: 46-year-old woman without comorbidities, who persisted with pain for seven months, without significant response to rheumatologic treatment.
17	Salazar, 2024, Mexico ^( 35 )^	To describe a case report of myalgia and arthralgia symptoms after COVID-19 ^†^ infection.	Case study.	N ^‡^ = 1, 75-year-old female. Patient without history of autoimmune or rheumatic disease. After drug treatment, complete recovery was achieved within six months of follow-up.
18	Khoja, 2024, United Kingdom ^( 36 )^	To investigate the clinical characteristics of recent-onset chronic pain in patients with Long COVID ^†^ and its impact on physical function, mood, and quality of life.	Cross-sectional study.	N ^‡^ = 30, 63% female; mean age 46.8 years. Pain was associated with central sensitization, elevated pro-inflammatory cytokines, weakness, reduced function, and decreased physical activity.
19	Akarsu, 2024, Turkey ^( 37 )^	To determine the frequency and characteristics of persistent pain in individuals diagnosed with COVID-19 ^†^ and compare them with those who recovered or did not experience persistent pain.	Cohort study.	N ^‡^ = 191, 51.8% male; mean age 41.4 years. Significant predominance of headaches in female patients (P ^§^ <0.001). Thirty-nine patients reported an exacerbation of their pre-COVID ^†^ headaches. Compared to other COVID-19 ^†^ , patients, those with persistent neuropathic pain characteristics were female (P ^§^ = 0.02), older (P ^§^ =0.02) and had higher incidence of symptoms such as sore throat (P ^§^ = 0.003) and headache (P ^§^ = 0.04).

*N = Number; ^†^COVID = Coronavirus Disease; ^‡^N = Study sample; ^§^P = Significance level

Among the 19 studies analyzed, it was observed that Long COVID manifests across all age groups, from children to older adults^(19-37)^. When analyzing the relationship between age group and the development of pain in Long COVID, one study found that adults who reported pain symptoms in Long COVID were younger than those who did not report pain (45 vs. 53 years)^(20)^; however, the opposite was observed in another study, in which adults who developed chronic pain, compared to those without, were older (50 vs. 42 years)^(29)^.

Regarding sex, in ten of the studies analyzed, the sample was predominantly female^(19,22,24-26,29-30,33-34,36)^. In terms of the relationship between gender and the development of pain in Long COVID, studies indicated that women were more likely to experience chronic pain^(29,37)^, and reported higher levels of pain intensity^(22,31)^.

With regard to comorbidities, one study found no association between their presence and the occurrence of pain^(20)^; however, another study showed that a higher Body Mass Index (BMI) was associated with longer durations of musculoskeletal symptoms, including chest pain, arthralgia, and myalgia^(21)^. Moreover, another study identified a higher prevalence of comorbidities among individuals with prolonged pain^(27)^.

Studies also demonstrated that, even after initiating treatment for Long COVID symptoms, some individuals continued to experience symptoms or did not achieve complete resolution^(19,34)^. In addition, studies reported that individuals with preexisting pain prior to COVID-19 infection experienced an exacerbation of their pain following the infection^(23,31,33,37)^.

Regarding pain location, as presented in Figure 4, the most frequently reported regions in the studies were: head^(20,24-25,28,30,32-33,37)^; thoracic^(19-23,27-28,34)^; neck^(22,27-31,34-35)^; back^(20,22-23,27-28,31,37)^; shoulder^(22-23,28,31,34-36)^; lower back^(21,24,27,29,31,36)^; hip^(21-22,31,34)^; hands^(23,29,31,34)^; knees^(22-23,31,34,36)^; legs^(28,30,34)^; joints^(22,29,31,35-36)^; thigh^(29,31)^; arms^(28,31)^; feet^(29,31)^; abdomen^(19,24)^ and only one study addressed ocular pain^(26)^.

**Figure 4-:** Description of pain according to location, frequency, duration, intensity, and quality. São Carlos, SP, Brazil, 2024

N*	**Location**	**Frequency and duration**	**Intensity**	**Quality**
1	Abdomen (upper and lower right quadrant)	Frequency: intermittent Duration: longer than three months	Not measured	Colic or burning
2	Back (17.0%), chest/thorax (14.2%), head (14.2%)	Frequency: not described Duration: not described	Scale used: Pain, Enjoyment, General Activity (0-10) Back: mean score 6.3 (moderate) Chest/thorax: mean score 3 (mild) Head: mean score 7.6 (severe)	Not described
3	Head, neck, back, lower back, hip, legs, chest/thorax	Frequency: not described Duration: not described	Not measured	Not described
4	Knees (18%), head (15%), neck (13%), shoulder (13%), back (9%)	Frequency: not described Duration: mean of 18.8 months	Scale used: Numerical Pain Rating Scale (0-10) Women: mean score 5.9 (moderate) Men: mean score 5.2 (moderate)	Not described
5	Generalized (22.7%), chest/thorax (19.9%), lower limbs (11.5%), cervical spine (8.5%), shoulders (8.5%), lumbar spine (7.9%), upper limbs (6.8%), wrist/elbow (5.7%), knees (5.7%), hips (2.8%)	Frequency: not described Duration: longer than 6 months	Not measured	Not described
6	Joints (7.6%), lower back (4.8%), head (3.2%), abdomen (2.0%)	Frequency: not described Duration: mean of 5.32 months	Not measured	Not described
7	Head (72.1%): diffuse 47.4%, frontoparietal 5.6%, frontal 5.1%, and parietal 4.2%	Frequency: 1–2 times/month (38.6%), 1–2 times/week (14.9%), more than 2 times/week (7.4%), daily (39.1%) Duration: not described	Scale used: Numerical Pain Rating Scale (0-10) Moderate (46.5%), mild (42.3%), and severe (11.1%)	Throbbing (59.1%), pressure (20.9%), mixed (6.0%), stabbing (5.1%), burning (1.9%)
8	Ocular (24.5%): bilateral 90.9%	Frequency: not described Duration: more than 14 days	Not measured	Not described
9	Neck, arm, back, waist	Frequency: not described Duration: mean of 4.38 months	Scale used: Visual Analog Scale for pain (0–10) Mean score 7.2 (severe)	Burning (23.2%), numbness (21.7%), tingling (14.5%), stinging (5.8%), freezing (1.4%)
10	Neck, shoulder, upper back, bilateral upper posterior arms, and lower posterior legs	Frequency: not described Duration: 12 months	Scale used: Pain Likert Scale (0-10) Score 6 (moderate)	Not described
11	Lower back (37.8%), joints (28.9%), neck (12.2%)	Frequency: not described Duration: not described	Scale used: Brief Pain Inventory (0-10) Mean score 5.12 (moderate)	Not described
12	Neck (69.1%), legs (68%), head (63.9%)	Frequency: not described Duration: mean of 104 weeks	Scale used: Brief Pain Inventory (0-10) Mean score 5.12 (moderate)	Not described
13	Arthralgia: shoulder (44.3%), knee (40.2%), elbow (26.8%), hip (22.7%), wrist (21.9%), foot/ankle (13.4%) Myalgia: neck/back/lower back (60.8%), calf (45.4%), arm (44.3%), forearm (38.1%), thigh (15.5%), anterior trunk (16.5%)	Frequency: not described Duration: mean of 4.0 months Overall score: 7 (severe) Women’s score: mean 7 (severe) Men’s score: mean 6 (moderate)	Scale used: Visual Analog Scale for pain (0-10)	Not described
14	Head (23.4%)	Frequency: not described Duration: not described	Not measured	Not described
15	Head (occipital 23.3%, bitemporal 22.8%, frontal 21.2%, diffuse 18.9%, unilateral 14.1%)	Frequency: daily (27.5%), 2–5x/week (44.1%), weekly (18.6%), monthly (9.8%) Duration: mean of 321.46 days	Scale used: Visual Analog Scale for pain (0-10) Score 5 (65.7% – moderate) and 8 (25.5% – severe)	Pressing (57.8%), throbbing (27.5%), burning (6.9%), mixed (7.8%)
16	Case 1: knees, shoulders, hands, back, chest/thorax, neck, jaw, wrist, knee, and ankle Case 2: knees, shoulders, ankles, hips, lower back, neck, and wrists	Frequency: not described Duration: More than 3 months (Case 1) More than 7 months (Case 2)	Not measured	Not described
17	Neck, shoulders, forearms	Frequency: not described Duration: mean of 6 months	Scale used: not specified in the study Classification: severe	Not described
18	Knees (70%), shoulders (63%), cervical spine (60%), and lumbosacral region (57%)	Frequency: intermittent (episodic) (10%), continuous (90%) Duration: mean of 519.1 days	Scale used: Brief Pain Inventory (0-10) Mean score 5.3 (moderate)	Dull and aching: 28 (93%) Sharp and stabbing: 2 (7%)
19	Head (29.8%): bilateral 80.7% Myalgia: limbs (54.5%), back (36.4%), generalized (9.1%)	Frequency: Headache: Less than 1 day/month (5.3%) 1–4 days/month (73.7%) 5–14 days/month (12.3%) More than 15 days (8.8%) Frequency of other sites: not described Duration: 1.5 years Headache: Mild (29.8%) Moderate (33.3%) Severe (36.9%) Scale used: not specified in the study Myalgia: Mean score 6.3	Scale used: classification according to the authors	Headache: Throbbing (47.4%) Pressing (38.6%) Burning (3.5%) Stabbing (5.3%)

*N = Number

With respect to frequency, pain may occur daily or in monthly episodes^(19,25,33-34,36-37)^. Regarding duration, some studies indicated that pain manifestations persisted for only a few months, whereas others reported persistence for more than one year^(19,22-24,26-28,30-31,33-37)^.

In studies addressing pain quality, the reported descriptors included colicky, burning, throbbing, pressing, stabbing, stinging, numbness, freezing, penetrating, or mixed^(19,25,27,33,36-37)^. As for headache characteristics, two studies showed that it may present as acute, stabbing, pressing, burning, throbbing, or mixed; it may also involve the entire head or a specific region^(25,33,37)^. To assess pain intensity, studies employed different scales, and findings indicated that pain ranged from mild to severe across the samples^(20,22,25,27-31,33-37)^.

## Discussion

In the analysis of the studies included in this review, it was found that pain as a symptom of Long COVID can affect individuals of all ages, with higher prevalence among women. Pain in Long COVID may involve different regions of the body; however, the most frequently reported sites in the studies analyzed were the thoracic region, head, neck, shoulder, lower back, and hip. Regarding pain quality, it may present in different forms depending on the site, such as colic, burning, throbbing, pressure, stabbing, stinging, numbness, freezing, penetrating, or mixed. Pain intensity ranged from mild to severe, with frequencies varying from daily to intermittent, and durations ranging from a few months to several years. This analysis also showed that individuals who experienced pain prior to COVID-19 infection may have their pain exacerbated following the viral infection.

The pathophysiology of pain in Long COVID is not yet fully understood; however, among the possible explanations is the wide diversity of symptoms manifested in the disease, stemming from the multisystemic nature of SARS-CoV-2 infection in the acute phase, which involves multiple organs and systems. Thus, the regions in which pain manifests may be related to multiorgan sequelae, namely the sites of tissue damage caused by viral invasion during the acute infection. These injuries may be considered the initial triggers of the symptoms^(38-39)^.

However, the persistence or emergence of symptoms after the acute phase of infection, which characterizes Long COVID, may be associated with mechanisms beyond tissue damage, such as the persistence of residual virus in the body In this context, one study demonstrated the persistence of SARS-CoV-2 in the small intestine four months after the onset of COVID-19 in half of the analyzed sample^(40)^. Thus, in addition to the respiratory tract, organs that express the angiotensin-converting enzyme 2 (ACE2) receptor—including body fluids such as saliva, urine, blood, tears, and semen—may serve as viral reservoirs, with viral persistence potentially triggering chronic inflammatory mechanisms^(38,41)^.

Accordingly, long-term inflammation after viral infection, resulting from a dysregulated immune response combined with individual risk factors, such as preexisting conditions—may contribute to loss of self-tolerance and hyperinflammation, as well as hyperactivation of thromboembolic and coagulation processes, multiorgan involvement, and autonomic nervous system dysfunction. Such dysfunction may sustain or perpetuate pathological processes, leading to the chronification of these symptoms in the affected organs and systems^(38-39)^.

In addition to the development of pain as a symptom of Long COVID, the literature indicates that individuals with preexisting pain prior to SARS-CoV-2 infection may experience exacerbation of pain after viral infection. In this context, one hypothesis is that virus-induced inflammatory mechanisms may contribute to hyperexcitability of the peripheral and central nervous systems through different pathways, thereby promoting the development of post-COVID pain or the aggravation of preexisting pain^(23,42)^.

Some factors have been identified in the literature as potential contributors to the risk of developing symptoms such as pain related to Long COVID, including hospitalization due to COVID-19, sex, age, and comorbidities. With respect to hospitalization, the studies included in this review did not provide comparisons of pain manifestation between individuals who were hospitalized for COVID-19 and those who were not.

However, analyses conducted in Spain and Brazil showed that the persistence of Long COVID symptoms was more prevalent in the hospitalized group than in those who did not require hospitalization, with myalgia reported more frequently among hospitalized individuals^(43-44)^.

It should be noted, however, that patients requiring intensive care may develop post–intensive care syndrome, which commonly occurs following prolonged critical illness. In addition, survivors may experience persistent inflammation, immunosuppression, and chronic organ dysfunction during hospitalization^(38-39,45)^. Accordingly, the literature has described that Long COVID symptoms may also be associated with post–intensive care syndrome, as well as with the combination of hospitalization-related sequelae, multiorgan involvement, and immunological dysfunction resulting from SARS-CoV-2 infection^(38)^.

Another risk factor frequently highlighted in the literature for general Long COVID symptoms, and particularly for pain manifestation, is female sex, which has been associated with longer symptom duration and greater functional limitations compared to males^(44-46)^.

Although only a few of the studies included in this review reported a direct association between being female and the development of Long COVID, it was observed that women made up the majority of the study samples. A study conducted in the United Kingdom indicated that women were at increased risk of developing general Long COVID symptoms compared to men^(47)^. Another review study showed that individuals of the female sex, compared to men, were more likely to develop myalgia in Long COVID^(46)^.

In this context, the literature highlights the existence of sexual dimorphism in most chronic pain conditions, including arthritis, migraine, and fibromyalgia, with higher prevalence in women than in men^(48)^. Among the hypotheses for sexual dimorphism in pain is the influence of sex hormones on pain sensitivity. Testosterone is suggested to exert antinociceptive and protective effects—namely, reducing pain sensitivity—whereas estradiol and progesterone have both pro- and antinociceptive effects. Accordingly, women with higher estradiol levels tend to exhibit reduced pain sensitivity and greater responsiveness to analgesics compared to women with lower estradiol levels. Moreover, pain sensitivity in women fluctuates across the menstrual cycle, with heightened sensitivity during the luteal phase, further underscoring the strong influence of sex hormones on pain^(49)^.

Regarding risk factors for the development of Long COVID, none of the studies included in this review examined the relationship between race and pain in Long COVID. However, particularly in the context of chronic pain, evidence indicates that racially marginalized groups disproportionately experience unrelieved symptoms. This is attributed to misconceptions about pain intensity during physician–patient interactions, unequal access to information, and inequitable access to healthcare services^(50)^.

The analyses in this review demonstrate that Long COVID can affect individuals across all age groups, from children to older adults^(19-34)^. Nevertheless, although advanced age may not appear to be a determinant for the development of Long COVID, evidence suggests that pain may manifest heterogeneously depending on the country of residence. In this context, an analysis of more than 100,000 participants from twenty countries showed that, among individuals aged 50 to 80 years, reported pain intensity increased with age in countries such as Korea, Israel, and Slovenia. By contrast, in countries such as the United States and Denmark, the association between pain intensity and older age was insignificant, with little variability in intensity^(51)^.

Thus, pain intensity may be influenced not only by chronological age but also by the conditions under which individuals age. In some high-income countries, people are regularly informed about potential treatment options for pain and, as a result, encounter fewer barriers to accessing pain assessment and treatment services^(51)^. Accordingly, pain assessment should include not only biological aspects but also the socioeconomic conditions to which individuals are exposed, as these can directly affect the perception of pain intensity.

Another point of divergence identified in the studies included in this review concerns the association between comorbidities and the risk of pain manifestation as a symptom of Long COVID. A study conducted in the United Kingdom reported that a baseline BMI in the overweight or obese range was associated with an increased risk of persistent symptoms, with individuals having a BMI greater than 30 kg/m^2^ compared to those with a BMI of 18.5–25 kg/m²^(47)^. Another analysis in Denmark showed a higher prevalence of pain among Long COVID patients with comorbidities^(52)^. The association between comorbidities and Long COVID symptoms may be attributed to the excessive release of cytokines during acute SARS-CoV-2 infection, which can exacerbate inflammatory mechanisms^(53)^.

Thus, although all individuals who have had COVID-19 are susceptible to developing Long COVID, some may be at greater risk. It is important to note that pain affects both physical and mental health, as well as quality of life. A study from Bangladesh demonstrated a significant inverse relationship between physical health status, quality of life, and the duration of Long COVID symptoms^(54)^.

Therefore, individuals with pain may not only endure physical suffering but also experience psychosocial symptoms and economic consequences, stemming from a lack of understanding by family members and health care professionals, as well as potential job loss due to persistent symptoms. Moreover, because chronic pain treatment is generally long term, its financial cost can pose a barrier, as management requires an interdisciplinary approach, while healthcare services often fail to provide comprehensive or timely care. Additionally, patients’ financial difficulties in covering treatment expenses may result in poor adherence or even discontinuation^(55)^.

In this context, it is important to emphasize that COVID-19 vaccination has been consistently identified as a protective factor against Long COVID^(45)^. A meta-analysis demonstrated that vaccinated individuals had a lower risk of developing any Long COVID symptom compared to those who were unvaccinated. The protective effect of vaccination was observed in patients who had received two doses, but not in those with only one dose. Furthermore, vaccination was effective against Long COVID both in individuals vaccinated prior to SARS-CoV-2 infection and in those vaccinated after infection^(56)^.

The literature also indicates that, even after initiating treatment for Long COVID–related pain, some individuals continue to experience symptoms, or these are not fully resolved. Thus, adherence to vaccination emerges as the most effective strategy to reduce the likelihood of developing severe manifestations of the disease, as well as the symptoms of Long COVID^(19,34,49)^.

It should also be emphasized that any clinical treatment approach must consider the specific and individual characteristics of pain. Accordingly, physical examination for pain assessment should include information on location, frequency, duration, intensity, and quality^(57)^. In this review, it was observed that the vast majority of studies focused on pain location and intensity, while frequency, duration, and quality were less frequently explored.

It should be noted that frequency helps in identifying persistence or recurrence of pain, whereas quality (including descriptors such as pulsating, burning, throbbing, among others) can contribute to understanding the nature of pain and plays a fundamental role in differentiating and diagnosing other painful clinical conditions^(58-59)^. Thus, the lack of a detailed approach to pain patterns and characteristics may limit a comprehensive understanding of the pain experience and its impact on the quality of life of individuals affected by this condition.

In addition to a thorough investigation of reported pain characteristics, the standardization of assessment using validated instruments is essential for monitoring clinical progression and optimizing care^(6,11,19-20)^. To support appropriate pain assessment across different age groups, instruments such as the numerical rating scale—where 0 corresponds to “no pain” and 10 to “worst possible pain”—can be used. The faces scale can be applied to individuals with speech difficulties or to children, in which pain intensity is rated based on illustrated facial expressions, ranging from a happy face (“no pain”) to the saddest face (“worst possible pain”)^(57)^.

It should also be emphasized that pain related to Long COVID persisting for more than three months can be classified as potential chronic pain. This type of pain may be considered an invisible disease and is often regarded as less important by health care professionals compared to other patient complaints, thereby minimizing its significance. In this regard, receiving a formal diagnosis of pain not only legitimizes the patient’s subjective experience but also enables adequate management of this symptom^(50)^. Given the incidence of pain in individuals with Long COVID and the biopsychosocial and economic impact that chronic pain can cause, it is essential that health care professionals acknowledge and value patients’ pain complaints.

Currently, care for individuals with Long COVID remains fragmented; however, recommendations indicate that this care should be organized in an integrated manner^(60)^. Early disease identification, continuous monitoring, health education, and the strengthening of self-care are fundamental, as they help prevent complications and ensure appropriate referrals when necessary. In this sense, integration across different levels of health care is essential for the effective management of pain, ensuring continuity of care for individuals with Long COVID^(59-62)^.

To date, there are no widely consolidated guidelines specifically for pain management in Long COVID. However, strategies that have been adopted include both pharmacological and non-pharmacological interventions, such as physiotherapy, graded exercise, occupational therapy, and psychological support. Therefore, integration among multidisciplinary teams is crucial, as effective pain management in Long COVID requires not only symptomatic control but also the prevention of potential chronification and the promotion of functional recovery^(12-14,61-62)^.

In this scenario, nursing plays a strategic role in the care of individuals with persistent pain in Long COVID, acting both in the continuous assessment of symptoms and in the implementation of interventions aimed at pain relief. The systematic use of validated pain measurement scales, combined with monitoring responses to therapies, allows for early adjustments to the therapeutic plan. Furthermore, nursing plays a central role in health education by providing guidance on self-care, adherence to therapies, prevention of exacerbations, and management of triggering factors. Qualified listening and the emotional support offered by nursing teams contribute to reducing the psychosocial impact of pain, thereby facilitating rehabilitation and improving quality of life^(12-14,62)^.

The limitations of this study include the restriction to publications available in Portuguese, English, and Spanish, which may have limited access to relevant evidence published in other languages. In addition, the choice of databases consulted was restricted, as different databases vary in journal coverage, indexing, and publication types.

Nevertheless, the results of this analysis have relevant implications for the advancement of scientific knowledge. Understanding the diversity of pain locations and characteristics in Long COVID underscores the complexity of this symptom and reinforces the need for developing more effective and individualized nursing care protocols.

## Conclusion

The findings of this scoping review suggest that pain in Long COVID can affect individuals of all ages, with higher prevalence among women. Pain, as a symptom of Long COVID, may develop in all regions of the body; however, it is most frequently observed in the head, chest, neck, shoulder, lower back, and hip. Its intensity ranges from mild to severe, with frequencies varying from daily to intermittent, and duration extending from a few months to several years, indicating the potential for symptom chronification.

Thus, the findings of this study on the clinical characteristics and patterns of pain may contribute to the development of specific guidelines for the management of this condition in individuals with Long COVID, enabling the differentiation of this pain from other painful conditions. It is also important to emphasize the need for preventive actions by encouraging adherence to COVID-19 vaccination, with the aim of reducing the likelihood of developing pain as a symptom of Long COVID, as well as lowering health care system costs through more accurate diagnosis of the disease.

Finally, this review highlights important gaps in the assessment of pain associated with Long COVID. The notable lack of detailed exploration of pain characteristics, with few studies exploring in detail aspects such as frequency, duration, and quality of pain in Long COVID, may contribute to changes in how pain is assessed in both research and clinical practice, helping to guide future investigations toward improving comprehensive approaches to pain assessment.

It is suggested that future studies include the analysis of individual variables that may influence the perception of pain as a symptom of Long COVID by health care professionals, such as the severity of acute COVID-19 infection, clinical examinations for the evaluation of inflammatory markers, presence of comorbidities, type of occupation, socioeconomic and environmental conditions of the region of residence, and COVID-19 vaccination status. These are variables that may influence health conditions and the long-term development of pain.

## Data Availability

All data generated or analysed during this study are included in this published article.
