# Supplementary material for: Pain in Long COVID: A scoping review of clinical characteristics and patterns of manifestation
Source: Rev Lat Am Enfermagem. 2025 Dec 1;33:e4777. doi: 10.1590/1518-8345.7836.4777 (PMC12685360; doi:10.1590/1518-8345.7836.4777)
Supplement: Translation

# Translation: Dor na COVID longa: uma revisão de escopo sobre características clínicas e padrões de manifestação*

**DOI:** 10.1590/1518-8345.7836.4778

**Authors:** Ana Cristina Ribeiro La Scaléa, Sílvia Carla da Silva André Uehara

**Affiliations:** 1 Universidade Federal de São Carlos, Departamento de Enfermagem, São Carlos, SP, Brasil. Universidade Federal de São Carlos Departamento de Enfermagem SP São Carlos Brasil; 2 Bolsista da Coordenação de Aperfeiçoamento de Pessoal de Nível Superior (CAPES), Brasil. Bolsista da Coordenação de Aperfeiçoamento de Pessoal de Nível Superior Brasil

**Keywords:** COVID-19, Síndrome de COVID-19 Pós-Aguda, Sinais e Sintomas, Dor, Dor Crônica, Saúde Pública

## Abstract

mapear as evidências científicas disponíveis sobre as características clínicas e padrões de manifestação da dor (localização, frequência, duração, intensidade e qualidade) em pessoas com a doença do novo coronavírus de longa duração (COVID longa).

revisão de escopo, com publicações de março de 2020 a junho de 2024, indexadas em quatro bases de dados. A seleção dos estudos foi realizada por dois revisores, de forma independente e cega, com extração dos dados por instrumento específico e análise descritiva.

incluídos 19 estudos que indicam que a dor afeta todas as faixas etárias, com maior prevalência entre mulheres, acometendo principalmente cabeça, pescoço, ombro, região lombar e quadril. A frequência varia de episódios diários a mensais, com duração superior a um ano em alguns casos. A intensidade varia de leve a intensa, e as características são diversas, incluindo descritores como queimação, pressão, cólica e dor latejante.

as características clínicas e padrões de manifestação da dor são amplos. No entanto, há escassez de estudos que abordem de forma detalhada as características da dor, bem como a influência de variáveis individuais. Tais achados devem orientar futuras investigações e práticas clínicas para uma avaliação mais abrangente e contextualizada da dor na COVID longa.

## Destaques

**(1)** Regiões que apresentam maior frequência da dor: tórax, cabeça, pescoço, ombro, lombar e quadril. **(2)** Intensidade pode variar de leve a intensa. **(3)** Duração de poucos meses a anos. **(4)** Exacerbação da dor preexistente após a infecção viral.

## Introdução

A doença do novo coronavírus (COVID-19) ocasionou mais de 280 milhões de casos da doença e 5 milhões de óbitos em todo o mundo^(1)^. Em maio de 2023, a Organização Mundial da Saúde (OMS) declarou o fim da emergência de saúde pública de interesse internacional imposta pela COVID-19; entretanto, o impacto nas condições de saúde das pessoas infectadas vai além da sobrevivência às formas graves da doença^(1-2)^.

Após a infecção viral aguda, começaram a ser observados relatos de sequelas pós-agudas de COVID-19, denominada também como COVID longa, caracterizada pelo desenvolvimento ou persistência de sintomas após 12 semanas da infecção aguda e que persistam por pelo menos dois meses^(3)^. Neste contexto, foi observado que a incidência da COVID longa pode variar de 10 a 70% dos casos da doença aguda, e pode se manifestar em pessoas que desenvolveram os quadros graves da COVID-19 até os mais leves^(4-5)^.

Embora a COVID longa não tenha seu mecanismo patogênico totalmente esclarecido, evidências científicas identificaram mais de duzentos sintomas, mostrando a natureza multissistêmica da nova doença, na qual alguns sintomas podem perdurar por semanas ou meses e outros, por tempo indeterminado^(3-4)^.

Dentre os sintomas listados na COVID longa, destaca-se a dor definida como “uma experiência sensitiva e emocional desagradável associada a danos reais ou potenciais”^(3-4,6)^. A dor, quando aguda, é um mecanismo de proteção que indica a presença de um estímulo potencialmente prejudicial; entretanto, qualquer dor que persista além do tempo normal de cicatrização do tecido, aproximadamente três meses, é descrita como dor crônica^(7-8)^.

É importante destacar que a dor crônica afeta aproximadamente 30% das pessoas em todo o mundo^(9)^ e espera-se que a dor como um sintoma da COVID longa incremente ainda mais essa taxa, visto que não há diretrizes únicas para o manejo dos pacientes com COVID longa. Nesse contexto, é fundamental avaliar as características da dor, como localização, intensidade, frequência e qualidade, a fim de determinar se ela é um sintoma da COVID longa ou se está relacionada a outras condições subjacentes^(5)^.

Adicionalmente, os profissionais de saúde devem considerar fatores como o sexo, idade, presença de comorbidades e o histórico de hospitalização devido à infecção pelo *Severe Acute Respiratory Syndrome –Coronavirus –*2 (SARS-CoV-2), a fim de auxiliar na identificação de pacientes com potencial para desenvolver a COVID longa^(10)^. A avaliação cuidadosa desses aspectos permite uma abordagem mais assertiva e personalizada, contribuindo para um diagnóstico mais preciso e uma gestão eficaz dos sinais e sintomas relacionados à COVID longa^(5)^.

Nesta conjuntura, a enfermagem desempenha um papel essencial na avaliação, no monitoramento e na implementação de intervenções eficazes para pessoas com COVID longa, especialmente no manejo da dor. Desta maneira, destaca-se a importância do diagnóstico de enfermagem, que orienta a tomada de decisões clínicas fundamentadas e individualizadas. A atuação do enfermeiro, pautada na sistematização da assistência de enfermagem, é fundamental para a identificação precoce do sintoma, o planejamento de cuidados e a promoção do conforto físico e emocional, contribuindo para a reabilitação integral do paciente^(11-14)^.

No contexto da COVID longa, a dor pode impactar diversos aspectos da vida dos indivíduos acometidos por essa condição, incluindo a redução da qualidade de vida e bem-estar social. Além disso, pessoas que apresentam dor crônica tendem a ter maior ausência do trabalho e a utilizar com mais frequência os recursos de saúde quando comparadas às pessoas sem dor crônica^(15)^.

Neste cenário, apesar do aumento significativo de estudos voltados às manifestações na COVID longa, predomina o enfoque em sintomas respiratórios, neurológicos e psicológicos^(3-5)^, enquanto a dor é frequentemente tratada como manifestação secundária, sem uma análise detalhada de suas características clínicas. Deste modo, observa-se uma lacuna quanto à compreensão das características da dor em pacientes com COVID longa, dificultando o desenvolvimento de estratégias terapêuticas direcionadas e baseadas em evidências.

O conhecimento das características clínicas e dos padrões da dor na COVID longa pode ajudar a distinguir a dor pós-COVID de outras condições dolorosas, bem como qualificar a prática profissional ao fornecer subsídios para decisões clínicas mais fundamentadas, favorecer a capacitação das equipes de saúde no reconhecimento precoce dessa manifestação e contribuir para o planejamento de ações interdisciplinares que atendam às demandas específicas dessa população^(3-5,10-14)^. Deste modo, este estudo tem como objetivo mapear as evidências científicas disponíveis sobre as características clínicas e padrões de manifestação da dor (localização, frequência, duração, intensidade e qualidade) em pessoas com a doença do novo coronavírus de longa duração (COVID longa).

## Método

### Desenho do estudo

Este estudo trata de uma revisão de escopo, estruturada com base no manual para síntese de evidências do *Joanna Briggs Institute* (JBI), sendo eles: a identificação da questão de pesquisa, identificação de estudos relevantes, seleção dos estudos, extração de dados, separação, sumarização e relatório de resultados e divulgação dos resultados^(16)^. O protocolo deste estudo está publicado na *Open Science Framework*: DOI 10.17605/OSF.IO/YVXDF.

### Identificação da pergunta de pesquisa

Para a construção da questão de pesquisa, foi utilizada uma estratégia que auxilia a identificar os tópicos-chave, recomendada em revisões de escopo, o método PCC, que representa uma mnemônica para P (População) (pessoas com dor como sintoma de COVID longa), C (Conceito) (dor) e C (Contexto) (COVID longa); definida como: Quais são as características clínicas e os padrões de manifestação (localização, frequência, duração, intensidade e qualidade) da dor em pacientes com COVID longa?

### Estratégia de pesquisa

Foram adotadas diferentes estratégias para localizar os artigos científicos, nas bases eletrônicas, a saber: *PubMed Central*, *Web of Science*, Scopus e Biblioteca Virtual em Saúde e a procura manual nas listas de referências dos artigos selecionados. O acesso às bases de dados ocorreu por meio do Portal de Periódicos da Coordenação de Aperfeiçoamento de Pessoal de Nível Superior (CAPES), a partir da identificação por meio da Comunidade Acadêmica Federada (CAFe), como forma de padronizar a coleta nessas bases.

As buscas foram conduzidas por meio de descritores e/ou termos alternativos, no idioma inglês, que constam no Descritores em Ciências da Saúde (DeCS) e no *Medical Subject Headings* (MeSH), conforme apresentado na Figura 1.

**Figura 1-:** Estratégia de busca nas bases de dados. São Carlos, SP, Brasil, 2024

**Base eletrônica**	**Estratégia empregada na busca**
*PubMed Central*	(Long COVID[Abstract] OR Post-Acute COVID-19 Syndrome[Abstract]) AND Pain[Abstract]
*Web of Science*	((AB=(“Long COVID”)) OR AB=(“Post-Acute COVID-19 Syndrome”)) AND AB=(Pain)
Biblioteca Virtual em Saúde	(ab:(long COVID)) OR (ab:(post-acute COVID-19 syndrome)) AND (ab:(pain)) AND instance:“lilacsplus”
Scopus	(ABS ( “Long COVID” ) OR ABS ( “Post-Acute COVID-19 Syndrome” ) AND ABS ( pain ) ) AND PUBYEAR > 2019 AND PUBYEAR < 2025 AND ( LIMIT-TO ( DOCTYPE , “ar” ) )

### Critérios de elegibilidade

Estabeleceram-se como critérios de inclusão estudos primários publicados nos idiomas português, inglês e espanhol entre março de 2020 e junho de 2024. Foram excluídos artigos duplicados, de opinião, editoriais, revisões, informações de *websites*, estudos cujos títulos e resumos não se delimitavam dentro do objetivo da investigação e artigos que não estavam disponíveis na íntegra e gratuitamente.

### Tratamento, análise e extração de dados

A busca dos artigos foi realizada nas bases de dados eletrônicas no mês de julho e o processo de seleção dos estudos ocorreu no período de agosto a outubro de 2024. Para auxiliar o processo de seleção dos estudos, após implementação da estratégia de busca em cada base de dados, as referências foram importadas para o aplicativo *webStArt (State of the Art through Systematic Review)*^(17)^, ferramenta de revisão desenvolvida pelo Laboratório de Pesquisa em Engenharia de *Software* (LaPES) da Universidade Federal de São Carlos (UFSCar). A seleção dos estudos ocorreu em dois níveis, a primeira seleção foi realizada por meio da leitura de títulos e resumos, seguida da leitura do artigo na íntegra. Os estudos elegíveis foram recuperados na íntegra e avaliados por dois pesquisadores. Em ambas as fases, as discrepâncias foram debatidas até chegar a um consenso, seguido da seleção definitiva.

A extração dos dados foi realizada por meio de um instrumento elaborado em formato de uma planilha desenvolvida no *Microsoft Excel*, contendo campos destinados a registrar informações relevantes dos estudos primários, tais como a identificação do estudo (autores, ano, país de publicação e objetivo), características metodológicas (tipo de estudo e tamanho da amostra) e principais resultados relacionados a características demográficas e clínicas e características relacionadas à dor como sintoma de COVID longa (localização, frequência, duração, intensidade e qualidade). Esse instrumento foi elaborado para garantir uniformidade e facilitar a organização dos dados, além de permitir posterior análise quantitativa e qualitativa dos resultados.

O processo de seleção dos estudos está apresentado em diagrama de fluxo e os resultados extraídos das publicações estão apresentados em figuras (quadros), no formato descritivo.

A elaboração desta revisão também aderiu às recomendações do *Preferred Reporting Items for Systematic Reviews and Meta-analyses Extension for Scoping Reviews* (PRISMA-ScR)^(18)^.

### Aspectos éticos

Com relação aos aspectos éticos da pesquisa, não houve discriminação na seleção dos artigos ou estudos, respeitando-se o critério de cegamento individual.

## Resultados

Foram identificados 1421 artigos nas bases de dados, desses, foram excluídos 809 por serem duplicatas, 515 após a leitura dos títulos e resumos e 78 após a leitura na íntegra. Assim, foram selecionados para o estudo 19 artigos que abordavam a dor como um sintoma de COVID longa (Figura 2).

**Figura 2-:** Fluxograma de referência: inclusão e exclusão dos artigos. São Carlos, SP, Brasil, 2024

As 19 (100%) publicações incluídas nesta revisão de escopo foram publicadas em inglês. Em relação aos países participantes dos estudos, destacam-se 5 (26,3%) estudos realizados nos Estados Unidos, 4 (21%) na Turquia, 3 (15,6%) na Espanha e 1 (5,3%) em cada um dos seguintes países: Irã, Indonésia, Chipre, Japão, Brasil, México e Reino Unido.

Quanto ao tipo dos estudos, destacam-se 9 (47,3%) estudos transversais, 5 (26,3%) coortes, 4 (21,1%) estudos de caso e 1 (5,3%) caso-controle. As informações relevantes de cada artigo selecionado, incluindo autores, ano de publicação, país onde o estudo foi realizado, desenho do estudo e amostra e os resultados principais, estão apresentados na Figura 3

**Figura 3-:** Descrição dos artigos, segundo autor, ano, local, objetivo, tipo de estudo e características demográficas e clínicas. São Carlos, SP, Brasil, 2024

**N** *	**Autor, ano e local**	**Objetivo**	**Tipo de estudo**	**Características demográficas e clínicas**
1	Arostegui, 2021, Estados Unidos ^( 19 )^	Descrever o relato de um caso de sintomas gastrointestinais em uma criança após três meses do diagnóstico de COVID-19 ^†^ .	Estudo de caso.	N ^‡^ = 1, 11 anos, sexo feminino. A criança iniciou com sintomas gastrointestinais durante a fase aguda da infecção por COVID-19 ^†^ , após três meses os sintomas ainda persistiam. Após três semanas de terapia, a dor abdominal não foi resolvida.
2	Savarraj, 2021, Estados Unidos ^( 20 )^	Caracterizar os resultados neurológicos de longo prazo após a COVID-19 ^†^ .	Estudo coorte.	N ^‡^ = 58, média de idade 49,2 anos e 54% do sexo masculino. Incluídas pessoas com histórico de hospitalização por COVID-19 ^†^ . O sintoma mais comum na amostra foi dor (60%). Aqueles que relataram dor eram significativamente mais jovens (45 versus 53 anos; P ^§^ < 0,05). Mulheres apresentaram maior frequência de dor, a diferença não foi estatisticamente significativa, e não houve variação quanto à etnia ou histórico médico prévio.
3	Azadvari, 2022, Irã ^( 21 )^	Avaliar a prevalência de diferentes sintomas musculoesqueléticos e seus fatores associados em pacientes com COVID ^†^ longa.	Estudo transversal.	N ^‡^ = 239, média de idade 37,96 anos; 69,9% do sexo feminino. Na amostra, mialgia (80,3%) e dor de cabeça (62,3%) foram os sintomas mais comuns. Índice de massa corpórea e os dias perdidos devido à COVID-19 ^†^ foram significativamente maiores em pacientes que apresentaram dor no peito, pernas, artralgia e mialgia por períodos mais longos (P ^§^ < 0,05). O sexo feminino foi associado a um maior risco de acamado devido à dor (P ^§^ = 0,037).
4	Peñas, 2022, Espanha ^( 22 )^	Identificar a correlação entre a extensão da dor e sintomas sensoriais, associados à sensibilização, variáveis cognitivas e psicológicas em sobreviventes de COVID-19 ^†^ com dor pós-COVID ^†^ .	Estudo transversal.	N ^‡^ = 146, média de idade 57,5 anos; 54,11% do sexo feminino. Incluídas pessoas com histórico de hospitalização por COVID-19 ^†^ . Quanto maior a extensão da dor, menor a intensidade da dor (P ^§^ =0,014). A associação entre a extensão da dor e a intensidade da dor foi significativa, mas ainda fraca, em homens (P ^§^ = 0,006), mas não em mulheres com dor pós-COVID ^†^ .
5	Peñas, 2022, Espanha ^( 23 )^	Investigar a associação entre biomarcadores sorológicos de gravidade da COVID-19 ^†^ na admissão hospitalar com sintomas de dor pós-COVID ^†^ em sobreviventes de COVID-19 ^†^ previamente hospitalizados.	Estudo de coorte.	N ^‡^ = 412, média de idade 62 anos; 53,9% do sexo masculino. A prevalência de dor foi de 42,7% e 36,2% em 6,8 e 13,2 meses após a alta hospitalar. Pacientes que desenvolveram dor pós-COVID ^†^ 6 meses após a alta hospitalar apresentaram um número maior de sintomas na admissão hospitalar e um número maior de comorbidades do que aqueles que não apresentaram dor pós-COVID ^†^ em seis meses. Entre os que desenvolveram dor pós-COVID ^†^ , 51,7% já tinham dor prévia, com exacerbação da dor em 16,4% dos casos.
6	Güven, 2022, Turquia ^( 24 )^	Identificar os preditores de COVID ^†^ longa realizando uma análise do quadro clínico, laboratorial e características demográficas de crianças com COVID-19 ^†^ .	Estudo coorte.	N ^‡^ = 500, média de idade 12,75 anos (COVID ^†^ positivo) e 12,50anos (grupo controle). Em ambos os grupos, 50,2% eram do sexo feminino. No total, 12,4% das crianças notaram sintomas de COVID ^†^ longa. Os sintomas foram significativamente mais comuns em pacientes que apresentavam sinais de infecção do trato respiratório superior no exame físico (P ^§^ = 0,025).
7	Mutiawati, 2022, Indonésia ^( 25 )^	Determinar as características da cefaleia em pacientes pós-COVID-19 ^†^ na Indonésia.	Estudo transversal.	N ^‡^ = 215, 45,6% tinham idade entre 30 e 39 anos; 69,8% do sexo feminino. 72,1% dos participantes sentiram dor de cabeça durante a hospitalização ou autoisolamento para COVID-19 ^†^ . Fatores como atividade física, ruído e luz forte, ou uma combinação de ambos, agravaram a dor de cabeça, enquanto repouso, analgésicos e uma combinação de repouso e analgésicos ajudaram a aliviar a dor.
8	McHarg, 2022, Estados Unidos ^( 26 )^	Caracterizar sistematicamente sintomas oculares encontrados na maioria dos indivíduos não hospitalizados com infecção por COVID-19 ^†^ .	Estudo transversal.	N ^‡^ = 229, média de idade 42,5 anos; 78,1% do sexo feminino. Dor nos olhos foi descrita em 24,5% da amostra. Sintomas oculares foram mais frequentes em indivíduos com histórico ocular prévio; apenas 13,3% procuraram oftalmologista, e, entre estes, 40,9% tiveram os sintomas atribuídos à COVID-19 ^†^ .
9	Topal, 2022, Turquia ^( 27 )^	Investigar a dor musculoesquelética prolongada como um sintoma de condição pós-COVID-19 ^†^ .	Estudo transversal.	N ^‡^ = 501, média de idade 39 anos e 51,3% eram do sexo masculino. Sessenta e nove participantes relataram dor prolongada pós-COVID ^†^ . Entre eles, 58% eram mulheres, sem diferenças significativas entre os sexos. A incidência de comorbidade geral ou a presença de uma ou mais condições adicionais concomitantes à COVID-19 ^†^ foi maior em pacientes com dor prolongada.
10	Zha, 2022, Estados Unidos ^( 28 )^	Descrever um relato de caso sobre dor miofascial após infecção por COVID-19 ^†.^	Estudo de caso.	N ^‡^ = 1, 59 anos, sexo masculino O paciente relatou incapacidade para realizar atividades da vida diária devido à dispneia persistente aos esforços, confusão mental e mialgia difusa. Após 12 meses, houve agravamento dos sintomas, associado ao aumento significativo do estresse psicossocial, que contribuiu para sua exacerbação.
11	Zis, 2022, Chipre ^( 29 )^	Estimar a prevalência da dor crônica em geral e da dor neuropática em particular, descrever as síndromes dolorosas mais comuns e identificar os determinantes da dor em uma população aleatória de sobreviventes da COVID-19 ^†^ .	Estudo transversal.	N ^‡^ = 90, média de idade 47,5 anos; 62,2% do sexo feminino. 63,3% dos participantes apresentavam dor crônica, sendo mais frequente em pessoas mais velhas (50 *versus* 42 anos) e mulheres. Após ajuste para idade e sexo, a dor de cabeça durante a COVID-19 ^†^ foi preditora significativa de dor neuropática, com risco 4,9 vezes maior.
12	Mateo, 2023, Espanha ^( 30 )^	Avaliar a qualidade de vida relacionada à saúde e as características da dor em pacientes com COVID ^†^ longa e comparar a localização da dor com aqueles que se recuperaram com sucesso da COVID-19 ^†^ .	Estudo de caso-controle.	N ^‡^ = 213, no grupo de COVID ^†^ longa, média de idade 44,99 anos; 75,26% do sexo feminino. A prevalência, intensidade e generalização da dor foram significativamente maiores em pessoas com COVID ^†^ longa em comparação a indivíduos recuperados e controles saudáveis.
13	Erden, 2023, Turquia ^( 31 )^	Investigar os sintomas musculoesqueléticos, tipo de dor e efeito na qualidade de vida em pacientes que apresentam dor após COVID-19 ^†^ .	Estudo transversal.	N ^‡^ = 97, média de idade 46,5 anos; 69,1% do sexo masculino. A pontuação média da escala visual analógica das mulheres, bem como o número de pacientes do sexo feminino que desenvolveram mialgia após a COVID-19 ^†^ foram significativamente maiores do que os dos homens (P ^§^ <0,05). Pessoas com dor musculoesqueléticas prévias, tiveram sua dor exacerbada após infecção por COVID-19 ^†^ .
14	Fujita, 2023, Japão ^( 32 )^	Esclarecer as características das dores de cabeça em pacientes com COVID ^†^ longa, concentrando-se na história e nas condições clínicas dos pacientes e conhecer o impacto das dores de cabeça na qualidade de vida desses pacientes.	Estudo coorte.	N ^‡^ = 482, média de idade 37 anos; 56% do sexo feminino. 23,4% dos participantes apresentavam dor de cabeça. Na fase aguda da COVID-19 ^†^ , a maioria dos casos de COVID ^†^ longa teve gravidade leve tanto no grupo cefaleia (88,5%) quanto no grupo sem cefaleia (82,7%).
15	Rodrigues, 2023, Brasil ^( 33 )^	Avaliar as características clínicas da cefaleia por COVID ^†^ longa.	Estudo transversal.	N ^‡^ = 102, 62,7% tinham acima de 39 anos; 81,37% do sexo feminino. A cefaleia na COVID ^†^ longa foi observada com mais frequência em mulheres com mais de 39 anos. Um total de 49,0% dos participantes possuía histórico prévio de dor de cabeça e relataram piora da dor na fase da COVID ^†^ prolongada.
16	Yadav, 2023, Estados Unidos ^( 34 )^	Descrição de relatos de caso de cinco pacientes que desenvolveram dor nas articulações várias semanas após a recuperação da infecção aguda por COVID-19 ^†^ .	Estudo de caso.	N ^‡^ = 5, sexo feminino, idade entre 19 e 61 anos. Três casos relatados no estudo apresentaram sintoma de dor inferior a um mês após a infecção por COVID-19 ^†^ ; desta forma, não foi considerado como COVID ^†^ longa. Primeiro caso: em mulher de 45 anos com obesidade mórbida e artrose, houve melhora de 70-80% dos sintomas após três meses de corticosteroides. Segundo caso: mulher de 46 anos, sem comorbidades, persistiu com dor por sete meses, sem resposta significativa ao tratamento reumatológico.
17	Salazar, 2024, México ^( 35 )^	Descrever um relato de caso de sintomas de mialgia e artralgias após infecção por COVID-19 ^†^ .	Estudo de caso.	N ^‡^ = 1, sexo feminino, 75 anos. Paciente sem histórico de doença autoimune ou reumática. Após tratamento medicamentoso, apresentou recuperação completa em seis meses de acompanhamento.
18	Khoja, 2024, Reino Unido ^( 36 )^	Investigar as características clínicas da dor crônica de início recente em pacientes com COVID ^†^ longa e seu impacto na função física, humor e qualidade de vida.	Estudo transversal.	N ^‡^ = 30, 63% eram do sexo feminino e a média de idade foi de 46,8 anos. A dor estava associada à sensibilização central, elevação de citocinas pró-inflamatórias, fraqueza, redução da função e da atividade física.
19	Akarsu, 2024, Turquia ^( 37 )^	Determinar a frequência e as características da dor persistente em indivíduos diagnosticados com COVID-19 ^†^ e compará-los com aqueles que se recuperaram ou não sentiram dor persistente.	Estudo de coorte.	N ^‡^ = 191, 51,8% eram do sexo masculino, média de idade 41,4 anos. Predominância significativa de cefaleias em pacientes do sexo feminino (P ^§^ <0,001). Trinta e nove pacientes relataram uma exacerbação de sua dor de cabeça anterior à infecção. Em comparação com outros pacientes com COVID-19 ^†^ , aqueles que apresentaram características de dor neuropática persistente foram do sexo feminino (P ^§^ =0,02), idade mais avançada (P ^§^ =0,02) e maior incidência de sintomas como dor de garganta (P ^§^ =0,003) e dor de cabeça (P ^§^ =0,04).

*N = Número; ^†^COVID = *Coronavirus Disease*; ^‡^N = Amostra do estudo; ^§^P = Nível de significância

Dentre os 19 estudos analisados, foi observado que a COVID longa se manifesta em todas as idades, da população infantil à idosa^(19-37)^. Ao analisar a relação entre faixa etária e o desenvolvimento de dor na COVID longa, um estudo evidenciou que pessoas adultas que relataram sintomas de dor na COVID longa eram mais jovens do que aquelas que não relataram dor (45 versus 53 anos)^(20)^; entretanto, o oposto foi observado em outro estudo, em que as pessoas adultas que desenvolveram dor crônica, comparadas aos indivíduos sem, eram mais velhas (50 versus 42 anos)^(29)^.

Quanto ao sexo, em dez dos estudos analisados, a amostra era composta predominantemente pelo sexo feminino^(19,22,24-26,29-30,33-34,36)^. Quanto à análise do sexo e ao desenvolvimento de dor na COVID longa, estudos indicaram que o sexo feminino esteve mais propenso à dor crônica^(29,37)^, bem como apresentaram níveis mais elevados de intensidade de dor^(22,31)^.

No que se refere à presença de comorbidades, destaca-se que um estudo não encontrou associação entre a presença de comorbidades e pessoas com ou sem dor^(20)^; por outro lado, outro estudo mostrou que um maior Índice de Massa Corpórea (IMC) foi associado a períodos mais longos quanto à manifestação de sintomas musculoesqueléticos, incluindo a dor torácica, artralgia e mialgia^(21)^. Ainda, em um outro estudo, foi identificado que a presença de comorbidades foi maior em pessoas com dor prolongada^(27)^.

Estudos também mostraram que, mesmo após iniciar algum tratamento para os sintomas de COVID longa, algumas pessoas permaneceram com os sintomas ou não foram eliminados totalmente^(19,34)^. Ainda, estudos apontaram que pessoas com dor anterior à infecção por COVID-19 tiveram sua dor exacerbada após a infecção^(23,31,33,37)^.

Na descrição da localização da dor, conforme apresentado na Figura 4, as regiões mais frequentes descritas nos estudos foram: cabeça^(20,24-25,28,30,32-33,37)^; torácica^(19-23,27-28,34)^; pescoço^(22,27-31,34-35)^; costas^(20,22-23,27-28,31,37)^; ombro^(22-23,28,31,34-36)^; lombar^(21,24,27,29,31,36)^; quadril^(21-22,31,34)^; mãos^(23,29,31,34)^; joelhos^(22-23,31,34,36)^; pernas^(28,30,34)^; articulações^(22,29,31,35-36)^; coxa^(29,31)^; braços^(28,31)^; pés^(29,31)^; abdome^(19,24)^ e apenas um estudo abordou a dor ocular^(26)^.

**Figura 4-:** Descrição da dor, segundo localidade, frequência, duração, intensidade e qualidade. São Carlos, SP, Brasil, 2024

N*	**Localização**	**Frequência e Duração**	**Intensidade**	**Qualidade**
1	Abdominal (quadrante superior e inferior direito)	Frequência: intermitente Duração: maior que três meses	Não mensurada	Cólica ou queimação
2	Costas (17,0%), peito (14,2%), cabeça (14,2%)	Frequência: não descrita Duração: não descrita	Escala utilizada: *Pain, Enjoyment, General Activity* (0-10) Costas: pontuação média 6.3 (moderado) Peito: pontuação média 3 (leve) Cabeça: pontuação média 7,6 (intensa)	Não descrita
3	Cabeça, pescoço, costas, lombar, quadril, pernas, peito	Frequência: não descrita Duração: não descrita	Não mensurada	Não descrita
4	Joelhos (18%), cabeça (15%), pescoço (13%), ombro (13%), costas (9%)	Frequência: não descrita Duração: média de 18,8 meses	Escala utilizada: Numérica de taxa de dor (0-10) Mulheres: pontuação média 5,9 (moderada) Homens: pontuação média 5,2 (moderada)	Não descrita
5	Generalizada (22,7%), tórax/peito (19,9%), extremidade inferior (11,5%), coluna cervical (8,5%), ombros (8,5%), coluna lombar (7,9%), extremidade superior (6,8%), punho/cotovelo (5,7%), joelho (5,7%), quadril (2,8%)	Frequência: não descrita Duração: maior que 6 meses	Não mensurada	Não descrita
6	Articulações (7,6%), lombar (4,8%), cabeça (3,2%), abdominal (2,0%)	Frequência: não descrita Duração: média 5,32 meses	Não mensurada	Não descrita
7	Cabeça (72,1%): difusa 47,4%, frontoparietal 5,6%, frontal 5,1%, e parietal 4,2%	Frequência: 1–2 vezes/mês (38,6%), 1–2 vezes/semana (14,9%), mais 2 vezes/semana (7.4%), diária (39.1%) Duração: não descrita	Escala utilizada: Numérica de taxa de dor (0-10) Moderada (46,5%), leve (42,3%) e intensa (11,1%)	Pulsátil (59,1%), pressão (20,9%), mista (6,0%), pontada (5,1%), ardente (1,9%)
8	Ocular (24,5%): bilateral 90,9%	Frequência: não descrita Duração: mais de 14 dias	Não mensurada	Não descrita
9	Pescoço, braço, costas, cintura	Frequência: não descrita Duração: média 4,38 meses	Escala utilizada: Numérica visual analógica da dor (0-10) Pontuação média 7,2 (intensa)	Queimação (23,2%), dormência (21,7%), formigamento (14,5%), ardência (5,8%), Congelamento (1,4%)
10	Pescoço, ombro, parte superior das costas, parte superior posterior bilateral dos braços e parte inferior posterior das pernas	Frequência: não descrita Duração: 12 meses	Escala utilizada: Likert de dor (0-10) Pontuação 6 (moderada)	Não descrita
11	Lombar (37,8%), articulações (28,9%), pescoço (12,2%)	Frequência: não descrita Duração: não descrita	Escala utilizada: *Brief Pain Inventory* (0-10) Pontuação média 5,12 (moderada)	Não descrita
12	Pescoço (69,1%), pernas (68%), cabeça (63,9%)	Frequência: não descrita Duração: média 104 semanas	Escala utilizada: *Brief Pain Inventory* (0-10) Pontuação média 5,12 (moderada)	Não descrita
13	Artralgia: ombro (44,3%), joelho (40,2%), cotovelo (26,8%), quadril (22,7%), pulso (21,9%), pé/tornozelo (13,4%) Mialgia: pescoço/costas/lombar (60,8%), panturrilha (45,4%), braço (44,3%), antebraço (38,1%), coxa (15,5%), frente do tronco (16,5%)	Frequência: não descrita Duração: média 4,0 meses	Escala utilizada: Numérica visual analógica de dor (0-10) Pontuação geral: 7 (intensa) Pontuação mulheres: média 7 (intensa) Pontuação homens: média 6 (moderada)	Não descrita
14	Cabeça (23,4%)	Frequência: não descrita Duração: não descrita	Não mensurada	Não descrita
15	Cabeça (occipital 23,3%, bitemporal 22,8%, frontal 21,2%, difusa 18,9%, unilateral 14,1%)	Frequência: diária (27,5%), 2–5x/semana (44,1%), Semanalmente (18,6%), mensal (9,8%) Duração: média 321,46 dias	Escala utilizada: Numérica visual analógica da dor (0-10) Pontuação 5 (65,7% -moderada) e 8 (25,5% - intensa)	Pressionante (57,8%), pulsante (27,5%), queimação (6,9%), mista (7,8%)
16	Caso 1: joelhos, ombros, mãos, costas, peito, pescoço, mandíbula, punho, joelho e tornozelo Caso 2: joelhos, ombros, tornozelos, quadris, lombar, pescoço e punhos	Frequência: não descrita Duração: Maior 3 meses (caso 1) Maior 7 meses (caso 2)	Não mensurada	Não descrita
17	Pescoço, ombros, antebraços	Frequência: não descrita Duração: média 6 meses	Escala utilizada: sem descrição no estudo Classificação: intensa	Não descrita
18	Joelhos (70%), ombros (63%), coluna cervical (60%) e região lombossacral (57%)	Frequência: intermitente (episódico)(10%), Contínuo (90%) Duração: média 519,1 dias	Escala utilizada: *Brief Pain Inventory* (0-10) Pontuação média 5,3 (moderada)	Dor surda e dolorosa 28 (93%) afiada e penetrante 2 (7%)
19	Cabeça (29,8%): bilateral 80,7%) Mialgia: extremidades (54,5%), costas (36,4%), generalizada (9,1%)	Frequência Cabeça: Menor 1 dia/mês (5,3%) 1–4 dias/mês (73,7%) 5–14 dias/mês (12,3%) Maior 15 dias (8,8%) Frequência de outras localidades: não descrita. Duração: 1,5 anos	Escala utilizada: classificação segundo os autores Cabeça: Leve (29,8%) Moderado (33,3%) Forte (36,9%) Escala utilizada: sem descrição no estudo Mialgia: Pontuação média 6,3	Cabeça: Pulsante (47,4%) pressionando (38,6%) ardente (3,5%) e esfaqueamento (5,3%)

*N = Número

Em relação à frequência, a dor pode se apresentar diariamente, bem como em episódios mensais^(19,25,33-34,36-37)^; já em relação ao tempo de duração da dor na COVID longa, alguns estudos indicaram que a manifestação da dor permanecia por poucos meses; já outras análises evidenciaram a persistência do sintoma por mais de um ano^(19,22-24,26-28,30-31,33-37)^.

Quanto aos estudos que abordaram as características da qualidade da dor, os descritores relatados foram como cólica, queimação, pulsátil, pressão, pontada, ardente, dormência, congelamento, penetrante ou mista^(19,25,27,33,36,37)^. Quanto às características da dor de cabeça, dois estudos evidenciaram que pode ser aguda, pontada, pressionando, queimação, pulsátil ou mista; ainda, pode acometer toda a cabeça ou uma região específica^(25,33,37)^. Para avaliar a intensidade da dor, os estudos utilizaram diferentes escalas, sendo observado que a dor variou de leve a intensa nas amostras^(20,22,25,27-31,33-37)^.

## Discussão

Na análise dos estudos incluídos nesta revisão, foi possível identificar que a dor como sintoma de COVID longa pode afetar todas as idades, sendo mais prevalente entre mulheres. A dor manifestada na COVID longa pode acometer regiões de todo o corpo, no entanto, os locais mais frequentes descritos nos estudos analisados foram a região do tórax, cabeça, pescoço, ombro, lombar e quadril. Quanto às características da qualidade da dor, pode se apresentar de formas distintas, a depender do local, como em cólica, queimação, pulsátil, pressão, pontada, ardente, dormência, congelamento, penetrante ou mista. A intensidade da dor variou de leve a intensa, com frequência diária a intermitente e a sua duração de poucos meses a anos. Esta análise evidenciou também que pessoas que possuíam dor antes da infecção por COVID-19, após a infecção viral, podem ter sua dor exacerbada.

A fisiopatologia da dor na COVID longa ainda não está totalmente esclarecida, no entanto, dentre as possíveis explicações, destaca-se a diversidade dos sintomas manifestados na doença devido à natureza multissistêmica da infecção pelo vírus SARS-CoV-2 na fase aguda, que envolve diversos órgãos e sistemas do organismo. Assim, a região na qual a dor se manifesta pode estar relacionada a sequelas multiorgânicas, ou seja, ao local onde ocorreram os danos teciduais devido à invasão viral na infecção na fase aguda. Estes danos podem ser considerados os geradores iniciais dos sintomas^(38-39)^.

No entanto, a persistência ou desenvolvimento de sintomas após a infecção da fase aguda, que caracteriza a COVID longa, pode estar relacionada a outros mecanismos além do dano tecidual, como a persistência de vírus residual no organismo. Neste cenário, um estudo evidenciou a persistência do SARS-CoV-2 no intestino delgado quatro meses após o início da COVID-19 em metade da amostra analisada^(40)^. Desta maneira, além do trato respiratório, órgãos que expressam o receptor da Enzima Conversora da Angiotensina 2 (ECA2), incluindo fluídos corporais, saliva, urina, sangue, lágrimas e sêmen, podem atuar como reservatório viral e a persistência do vírus pode desencadear mecanismos inflamatórios crônicos^(38,41)^.

Sendo assim, mecanismos de inflamação de longa duração após a infecção viral, resultantes da resposta imunológica desregulada, além da associação de fatores de risco do indivíduo infectado, como a presença de doenças preexistentes, podem contribuir para a perda de autotolerância e hiperinflamação, além de hiperativação de processos tromboembólicos/coagulantes, multiorgânicos e nervoso autônomo. Desta forma, a disfunção presente no organismo pode manter ou perpetuar os processos patológicos, levando à cronificação desses sintomas nos órgãos e sistemas afetados^(38-39)^.

Além do desenvolvimento da dor como um sintoma da COVID longa, a literatura aponta que pessoas com dor anterior à infecção do vírus SARS-CoV-2, após a infecção viral, podem ter a dor exacerbada. Neste cenário, uma hipótese seria que os mecanismos inflamatórios do vírus podem contribuir para a hiperexcitabilidade dos sistemas nervosos periférico e central por meio de diferentes vias, promovendo o desenvolvimento de dor pós-COVID ou o agravamento da dor^(23,42)^.

Alguns fatores têm sido apontados pela literatura como potencializadores do risco para o desenvolvimento de sintomas como a dor relacionada à COVID longa, como necessidade de hospitalização em decorrência da COVID-19, sexo, idade e comorbidades. No que se refere à hospitalização, nos estudos selecionados, não foram apresentadas comparações na manifestação da dor entre pessoas hospitalizadas ou não por COVID-19.

Por outro lado, análises na Espanha e no Brasil mostraram que a persistência dos sintomas de COVID longa foi mais prevalente no grupo hospitalizado do que no grupo de pessoas sem necessidade de hospitalização, sendo que a mialgia foi relatada com mais frequência no grupo hospitalizado^(43-44)^.

No entanto, é necessário ponderar que pessoas que necessitaram de hospitalizações em cuidados intensivos podem apresentar alguma síndrome de cuidados pós-intensivos, que geralmente ocorre após doenças críticas prolongadas. Além disso, os sobreviventes podem experimentar, durante a hospitalização, processos de inflamação persistente, imunossupressão e disfunção crônica de órgãos^(38-39,45)^. Desta maneira, a literatura tem descrito que sintomas da COVID longa também podem estar associados à síndrome dos cuidados intensivos, bem como uma combinação da síndrome resultante da hospitalização acrescida de sequelas multiorgânicas e disfunções imunológicas decorrentes da infecção pelo SARS-CoV-2^(38)^.

Outro fator de risco frequentemente apontado pela literatura em sintomas gerais de COVID longa, mas especialmente na manifestação da dor, é o sexo feminino, associado ao aumento da duração dos sintomas e limitações funcionais quando comparados aos indivíduos do sexo masculino^(44-46)^.

Embora poucos estudos selecionados nesta revisão tenham apresentado em suas análises associação do sexo feminino ao desenvolvimento da COVID longa, foi observado que o sexo feminino compunha a maioria das amostras. Um estudo no Reino Unido apontou que as mulheres apresentavam risco aumentado para o desenvolvimento de sintomas gerais de COVID longa em comparação aos homens^(47)^. Em outro estudo de revisão, foi apresentado que pessoas do sexo feminino, em comparação ao sexo masculino, tiveram uma maior probabilidade de desenvolver mialgia na COVID longa^(46)^.

Neste cenário, a literatura aponta a existência de dimorfismo sexual na maioria das condições de dor crônica, incluindo artrite, enxaqueca e fibromialgia, com as mulheres apresentando maior prevalência do que os homens^(48)^. Dentre as hipóteses para o dimorfismo sexual na dor, são descritas a influência dos hormônios sexuais na sensibilidade à dor. Desta maneira, sugere-se que a testosterona seja antinociceptiva e protetora, ou seja, diminui a sensibilidade à dor, enquanto o estradiol e a progesterona provocam efeitos pró e antinociceptivos. Logo, as mulheres com altos níveis de estradiol apresentam diminuição da sensibilidade à dor e aumento da efetividade dos analgésicos em comparação às mulheres com baixos níveis de estradiol. Ainda, a sensibilidade à dor de mulheres altera-se ao longo do ciclo menstrual, com sensibilidade aumentada durante a fase lútea, indicando uma forte influência dos hormônios sexuais na dor^(49)^.

Ainda, sobre os fatores de risco para o desenvolvimento da COVID longa, nos estudos selecionados nesta revisão, não foram identificadas abordagens da raça e a dor na COVID longa. Entretanto, especialmente na dor crônica, aponta-se que grupos racialmente marginalizados sofrem desproporcionalmente de sintomas não aliviados como resultado de percepções errôneas sobre a intensidade da dor durante a interação médico-paciente, acesso desigual à informação e aos cuidados de saúde^(50)^.

As análises desta revisão mostram que a COVID longa pode acometer todas as idades, desde a população infantil à idosa^(19-34)^. No entanto, embora a idade avançada possa aparentemente não ser um determinante para o desenvolvimento da COVID longa, há indícios de que a dor pode se apresentar de forma heterogênea segundo o país de moradia. Nesse contexto, uma análise com mais de cem mil participantes oriundos de vinte países mostrou que, em pessoas entre 50 e 80 anos, em países como a Coreia, Israel e Eslovênia, a intensidade da dor relatada se elevou conforme a idade também aumentava. Entretanto, em países como os Estados Unidos e Dinamarca, a associação entre a intensidade da dor associada ao aumento da idade foi insignificante, com pouca variabilidade na intensidade^(51)^.

Assim, a intensidade da dor não estaria relacionada apenas à idade cronológica, mas às condições em que as pessoas envelhecem. Em alguns países de rendimento elevado, as pessoas são constantemente informadas sobre potenciais opções de tratamento para a dor e, portanto, enfrentam menos barreiras no acesso aos serviços para avaliação e tratamento^(51)^. Desta forma, destaca-se a importância de incluir na avaliação da dor, além dos aspectos biológicos, as condições socioeconômicas às quais as pessoas estão expostas, o que pode influenciar diretamente a percepção da intensidade da dor.

Outro ponto de divergência encontrado nos estudos selecionados nesta revisão diz respeito à associação de comorbidades como um fator de risco para a manifestação da dor como um sintoma da COVID longa. Um estudo no Reino Unido descreveu que o IMC basal na faixa de sobrepeso ou obesidade foi associado a um risco aumentado de sintomas persistentes, com aqueles que tinham um IMC superior a 30 kg/m^2^ em comparação com aqueles com um IMC de 18,5–25 kg/m^2(47)^. Outra análise na Dinamarca mostrou uma maior prevalência de dor entre pacientes com COVID longa em pessoas com comorbidades^(52)^. A relação da presença de comorbidades e sintomas de COVID longa pode ser atribuída à liberação excessiva de citocinas na infecção aguda pelo SARS-CoV-2, que pode agravar os mecanismos inflamatórios^(53)^.

Assim, embora todas as pessoas que tiveram COVID-19 estejam suscetíveis ao desenvolvimento da COVID longa, algumas pessoas podem ter um risco aumentado para a doença. Ressalta-se que a dor afeta a saúde física e mental das pessoas, além de impactar a qualidade de vida. Estudo em Bangladesh mostrou uma relação inversa significativa entre o estado de saúde física, qualidade de vida e duração de sintomas de COVID longa^(54)^.

Logo, pessoas com dor, além do sofrimento físico, podem apresentar sintomas psicossociais e até mesmo impacto econômico, uma vez que pode haver uma falta de compreensão da família e dos profissionais de saúde, bem como até perdas de trabalho em decorrência dos sintomas. Ainda, visto que o tratamento da dor crônica geralmente é a longo prazo, o custo financeiro para geri-la pode se constituir como uma barreira, uma vez que exige um manejo interdisciplinar e os serviços de saúde muitas vezes não oferecem toda a assistência necessária ou em tempo hábil, e as dificuldades financeiras do paciente para financiar o tratamento podem resultar na baixa adesão ou desistência^(55)^.

Nesse contexto, é importante destacar que a vacinação contra a COVID-19 tem sido apontada fortemente como um fator protetor de COVID longa^(45)^. Uma metanálise mostrou que pessoas vacinadas tiveram um risco menor de desenvolver qualquer sintoma de COVID longa em comparação com pessoas não vacinadas. Em comparação com pacientes que não foram vacinados, a vacinação mostrou seu efeito protetor em pacientes vacinados com duas doses, mas não com uma dose. Além disso, a vacinação foi eficaz contra COVID longa em pacientes vacinados antes da infecção por SARS-CoV-2 ou vacinados após a infecção por SARS-CoV-2^(56)^.

A literatura apresenta que, mesmo após iniciar algum tratamento para a dor na COVID longa, algumas pessoas permanecem com os sintomas ou estes não são eliminados totalmente. Assim, ressalta-se que a adesão à vacinação se apresenta como a melhor alternativa para reduzir as chances de desenvolver sintomas graves da doença, bem como os sintomas da COVID longa^(19,34,49)^.

Destaca-se ainda que qualquer abordagem quanto ao tratamento clínico deve considerar as características específicas e individuais de dor. Desta maneira, o exame físico para avaliação da dor deve abordar as informações sobre localização, frequência, duração, intensidade e qualidade^(57)^. Nesta análise, foi possível observar que a grande maioria dos estudos se concentra na localização e intensidade da dor, sendo a frequência, duração e qualidade variáveis menos exploradas.

Destaca-se que a frequência auxilia na identificação em relação à persistência ou recorrência da dor, enquanto a qualidade, dentre elas a descrição como pulsante, queimação, latejante, entre outros, pode contribuir para a compreensão sobre a natureza da dor e desempenha um papel fundamental na diferenciação e diagnóstico de outras condições clínicas dolorosas^(58-59)^. Assim, a ausência na abordagem de padrões e características detalhadas pode limitar a compreensão completa da experiência da dor e seu impacto na qualidade de vida das pessoas acometidas por essa condição.

Além de uma investigação detalhada das características da dor referida, a padronização da avaliação por instrumentos validados contribui para monitorar a evolução clínica e otimizar o cuidado^(6,11,19-20)^. Para auxiliar na avaliação adequada da dor, nas diferentes faixas etárias, existem instrumentos como a escala de avaliação numérica em que a 0 corresponde à classificação “sem dor” e a 10 à “dor máxima”; já a escala de faces, pode ser utilizada em pessoas com alguma dificuldade na fala ou em crianças, a pessoa classifica a intensidade da dor com base na face desenhada, sendo que à expressão de felicidade corresponde a classificação “sem dor” e à expressão de máxima tristeza corresponde a classificação “dor máxima”^(57)^.

Ressalta-se que a dor decorrente da COVID longa que persista por mais de três meses se enquadra como potencial dor crônica, podendo ser considerada uma doença invisível e, muitas vezes, vista como de menor importância pelos profissionais de saúde quando comparadas a outras queixas dos pacientes, tendo assim seu sintoma minimizado. Posto isso, receber um diagnóstico formal de dor, além de legitimar a experiência subjetiva do paciente, também possibilita a oportunidade de manejo adequado desse sintoma^(50)^. Neste cenário, dada a incidência da dor em pessoas com COVID longa e o impacto biopsicossocial e econômico que a dor crônica pode causar, torna-se essencial a valorização da queixa de dor pelos profissionais de saúde.

Atualmente, observa-se uma fragmentação na assistência às pessoas com COVID longa, no entanto, a recomendação é que esta seja organizada de forma integrada^(60)^. A identificação precoce da doença, o monitoramento contínuo, a educação em saúde e o fortalecimento do autocuidado são fundamentais, prevenindo complicações e garantindo o encaminhamento adequado quando necessário. Desta maneira, a integração entre os diferentes níveis de atenção à saúde é essencial para a gestão eficaz da dor, garantindo a continuidade do cuidado das pessoas com COVID longa^(59-62)^.

Até o momento, não há diretrizes amplamente consolidadas específicas para o manejo da dor na COVID longa, no entanto, dentre as estratégias adotadas estão as intervenções farmacológicas tradicionais e não farmacológicas, que incluem, por exemplo, a fisioterapia, prática de exercícios graduais, terapia ocupacional e suporte psicológico. Deste modo, a integração entre equipes multiprofissionais é essencial, pois o manejo eficaz da dor na COVID longa requer não apenas controle sintomático, mas também prevenção de potencial cronificação e recuperação funcional^(12-14,61-62)^.

Neste cenário, a enfermagem exerce papel estratégico no cuidado de pessoas com dor persistente na COVID longa, atuando tanto na avaliação contínua dos sintomas quanto na implementação de intervenções orientadas para o alívio da dor. A aplicação sistemática de escalas validadas para mensuração da dor, aliada ao monitoramento da resposta às terapias instituídas, permite ajustes precoces no plano terapêutico. Além disso, a enfermagem tem papel central na educação em saúde, fornecendo orientações sobre autocuidado, adesão às terapias, prevenção de exacerbações e manejo de fatores desencadeantes. A escuta qualificada e o suporte emocional oferecidos pela equipe de enfermagem contribuem para reduzir o impacto psicossocial da dor, favorecendo a reabilitação e a melhora da qualidade de vida^(12-14,62)^.

Consideram-se como limitações deste estudo a inclusão de publicações exclusivamente nos idiomas português, inglês e espanhol, o que pode ter restringido o acesso a evidências relevantes em outros idiomas, e a escolha restrita das bases de dados consultadas, uma vez que diferentes bases apresentam variações na cobertura e indexação de periódicos e tipos de publicações.

Entretanto, os resultados desta análise apresentam implicações relevantes para o avanço do conhecimento científico nas áreas da saúde e da enfermagem. A compreensão da diversidade na localização e nas características da dor na COVID longa, que se manifesta de forma variada, reforça a complexidade desse sintoma e a necessidade do desenvolvimento de protocolos de atendimento mais eficazes e individualizados na prática de enfermagem.

## Conclusão

Os resultados desta revisão de escopo sugerem que a dor na COVID longa pode afetar todas as idades, sendo mais prevalente em mulheres. A dor, como um sintoma da COVID longa, pode se desenvolver em todas as regiões do corpo, no entanto, em algumas regiões, como a cabeça, tórax, pescoço, ombro, lombar e quadril, são mais frequentemente observadas. A intensidade pode variar de leve a intensa, com frequência diária à intermitente, e a duração pode ser de poucos meses a anos, sugerindo potencial cronificação dos sintomas.

Deste modo, os achados deste estudo sobre as características clínicas e os padrões da dor podem contribuir para a elaboração de diretrizes específicas para o manejo dessa condição em pessoas com COVID longa, permitindo a diferenciação dessa dor de outras condições dolorosas. Destaca-se ainda a necessidade de ações preventivas por meio do incentivo a adesão à vacinação contra a COVID-19 com a finalidade de reduzir as chances do desenvolvimento da dor como um sintoma da COVID longa, bem como reduzir os custos financeiros dos sistemas de saúde ao realizar um diagnóstico mais assertivo da doença.

Por fim, a partir desta revisão, destacam-se importantes lacunas quanto à abordagem da avaliação da dor relacionada à COVID longa. A ausência significativa da inclusão das características da dor, com poucos estudos explorando de forma detalhada aspectos como frequência, duração e qualidade da dor na COVID longa, pode contribuir para mudanças na forma como a dor é avaliada em pesquisas e práticas clínicas, auxiliando a orientar futuras investigações para melhorar a avaliação global da dor.

Sugere-se que, em estudos futuros, sejam incluídas a análise de variáveis individuais que possam influenciar a percepção da dor como um sintoma de COVID longa pelos profissionais da saúde, como a gravidade da infecção aguda por COVID-19, exames clínicos para a avaliação de indicadores inflamatórios, presença de comorbidades, tipo de ocupação, condições socioeconômicas e ambientais da região de moradia e situação vacinal contra a COVID-19, variáveis que podem influenciar as condições de saúde e o desenvolvimento da dor a longo prazo.

## Data Availability

Todos os dados gerados ou analisados durante este estudo estão incluídos neste artigo publicado.

# Translation: Dolor en el COVID persistente: revisión exhaustiva de las características clínicas y patrones de manifestación*

**DOI:** 10.1590/1518-8345.7836.4776

**Authors:** Ana Cristina Ribeiro La Scaléa, Sílvia Carla da Silva André Uehara

**Affiliations:** 1 Universidade Federal de São Carlos, Departamento de Enfermagem, São Carlos, SP, Brasil. Universidade Federal de São Carlos Departamento de Enfermagem SP São Carlos Brasil; 2 Becaria de la Coordenação de Aperfeiçoamento de Pessoal de Nível Superior (CAPES), Brasil. Becaria de la Coordenação de Aperfeiçoamento de Pessoal de Nível Superior Brasil

**Keywords:** COVID-19, Síndrome Post Agudo de COVID-19, Signos y Síntomas, Dolor, Dolor Crónico, Salud Pública

## Abstract

mapear las pruebas científicas disponibles sobre las características clínicas y los patrones de manifestación del dolor (localización, frecuencia, duración, intensidad y calidad) en personas con la enfermedad del nuevo coronavirus de larga duración (COVID prolongado).

revisión de alcance de publicaciones de marzo de 2020 a junio de 2024, indexadas en cuatro bases de datos. La selección de estudios fue realizada de forma independiente y ciega por dos revisores, con extracción de datos mediante un instrumento específico y análisis descriptivo.

se incluyeron diecinueve estudios y se advirtió que el dolor afecta a todos los grupos de edad, con mayor prevalencia entre mujeres, principalmente en regiones como cabeza, cuello, hombro, zona lumbar y cadera. La frecuencia varía de episodios diarios a mensuales, con una duración de más de un año en algunos casos. La intensidad varía de leve a grave/intensa, y las características son diversas, incluyendo descriptores como ardor, presión, cólico y dolor punzante.

las características clínicas y patrones de manifestación del dolor son amplios. Sin embargo, cabe señalar la escasez de estudios que aborden en detalle las características del dolor y la influencia de variables individuales. Tales hallazgos deben orientar futuras investigaciones y prácticas clínicas hacia una evaluación más integral y contextualizada del dolor en el COVID prolongado.

## Destacados

**(1)** Regiones que presentan mayor frecuencia de dolor: tórax, cabeza, cuello, hombro, zona lumbar y cadera. **(2)** La intensidad puede variar de leve a grave o intensa. **(3)** La duración puede extenderse de pocos meses hasta años. **(4)** Exacerbación del dolor preexistente después de una infección viral.

## Introducción

La enfermedad por el nuevo coronavirus (COVID-19) ha causado más de 280 millones de casos y 5 millones de muertes en todo el mundo^(1)^. En mayo de 2023, la Organización Mundial de la Salud (OMS) declaró el fin de la emergencia de salud pública de interés internacional causada por el COVID-19; sin embargo, el impacto en la salud de las personas infectadas va más allá de la supervivencia a las formas graves de la enfermedad^(1-2)^.

Tras la infección viral aguda, comenzaron a observarse reportes de secuelas post-agudas de COVID-19, también llamado COVID prolongado o persistente, caracterizado por la aparición o persistencia de síntomas después de 12 semanas de infección aguda y durante al menos dos meses^(3)^. En este contexto, se observó que la incidencia de COVID persistente puede variar entre el 10% y el 70% de los casos de enfermedad aguda, y puede manifestarse tanto en personas que desarrollaron COVID-19 grave como en casos más leves^(4-5)^.

Aunque el mecanismo patogénico del COVID prolongado no se comprende completamente, la evidencia científica ha identificado más de doscientos síntomas, lo que demuestra la naturaleza multisistémica de la nueva enfermedad, en la que algunos síntomas pueden durar semanas o meses y otros prolongarse por un período indefinido^(3-4)^.

Entre los síntomas del COVID persistente, destaca el dolor, definido como “una experiencia sensorial y emocional desagradable asociada a daños reales o potenciales”^(3-4,6)^. El dolor, cuando es agudo, es un mecanismo de protección que indica la presencia de un estímulo potencialmente dañino; sin embargo, cualquier dolor que persista más allá del tiempo normal de cicatrización del tejido, aproximadamente tres meses, se describe como dolor crónico^(7-8)^.

Es importante destacar que el dolor crónico afecta aproximadamente al 30% de la población mundial^(9)^, y se prevé que el dolor como síntoma de COVID persistente aumente aún más esta tasa, ya que no existen directrices únicas para el manejo de pacientes con COVID persistente. En este contexto, es fundamental evaluar las características del dolor, como la localización, la intensidad, la frecuencia y la calidad, para determinar si se trata de un síntoma de COVID persistente o está relacionado con otras afecciones subyacentes^(5)^.

Además, los profesionales de la salud deben considerar factores como el sexo, la edad, la presencia de comorbilidades y el historial de hospitalización debido a la infección por el *Severe Acute Respiratory Syndrome –Coronavirus –* 2 (SARS-CoV-2) para ayudar a identificar a los pacientes con potencial de desarrollar COVID prolongado^(10)^. La evaluación cuidadosa de estos aspectos permite un abordaje más asertivo y personalizado, contribuyendo a un diagnóstico más preciso y un manejo efectivo de los signos y síntomas relacionados con el COVID prolongado^(5)^.

En este contexto, la enfermería desempeña un papel esencial en la evaluación, el seguimiento y la implementación de intervenciones eficaces para personas con COVID persistente, especialmente en el manejo del dolor. Por lo tanto, se destaca la importancia del diagnóstico de enfermería, ya que orienta la toma de decisiones clínicas informadas e individualizadas. La actuación del enfermero, basada en la sistematización de los cuidados de enfermería, es esencial para la identificación temprana de síntomas, la planificación de los cuidados y la promoción del bienestar físico y emocional, contribuyendo así a la rehabilitación integral del paciente^(11-14)^.

En el contexto del COVID prolongado, el dolor puede afectar varios aspectos de la vida de las personas afectadas, incluyendo una reducción de la calidad de vida y del bienestar social. Además, las personas con dolor crónico tienden a tener más ausencias laborales y a utilizar los recursos sanitarios con mayor frecuencia que quienes no presentan dolor crónico^(15)^.

En este contexto, a pesar del aumento significativo de estudios centrados en las manifestaciones del COVID persistente, la atención se centra predominantemente en los síntomas respiratorios, neurológicos y psicológicos^(3-5)^, mientras que el dolor suele tratarse como una manifestación secundaria, sin un análisis detallado de sus características clínicas. Por lo tanto, existe una brecha en la comprensión de las características del dolor en pacientes con COVID persistente, lo que dificulta el desarrollo de estrategias terapéuticas específicas y basadas en evidencia.

Comprender las características clínicas y los patrones de dolor del COVID persistente puede ayudar a distinguir el dolor Post-COVID de otras afecciones dolorosas, así como a mejorar la práctica profesional al brindar apoyo para la toma de decisiones clínicas más informadas, promover la capacitación de los equipos de atención médica en el reconocimiento temprano de esta manifestación y contribuir a la planificación de acciones interdisciplinarias que satisfagan las necesidades específicas de esta población^(3-5,10-14)^. En ese marco, este estudio tiene como objetivo mapear la evidencia científica disponible sobre las características clínicas y los patrones de manifestación del dolor (ubicación, frecuencia, duración, intensidad y calidad) en personas con enfermedad por coronavirus de larga duración (COVID persistente).

## Método

### Diseño del estudio

Este estudio es una revisión exploratoria, estructurada según el manual de síntesis de evidencia del *Joanna Briggs Institute* (JBI), que incluye: identificación de la pregunta de investigación, identificación de estudios relevantes, selección de estudios, extracción de datos, separación, resumen y presentación de resultados, y difusión de los mismos^(16)^. El protocolo de este estudio está publicado en el *Open Science Framework*: DOI 10.17605/OSF.IO/YVXDF.

### Identificación de la pregunta de investigación

Para construir la pregunta de investigación se utilizó una estrategia que ayuda a identificar temas clave, recomendada en revisiones de alcance: el método PCC, que representa un mnemónico para P (Población) (personas con dolor como síntoma de COVID prolongado), C (Concepto) (dolor) y C (Contexto) (COVID prolongado); definido como: ¿Cuáles son las características clínicas y los patrones de manifestación (ubicación, frecuencia, duración, intensidad y calidad) del dolor en pacientes con COVID prolongado?

### Estrategia de investigación

Se adoptaron diferentes estrategias para localizar artículos científicos en bases de datos electrónicas, a saber: *PubMed Central*, *Web of Science*, Scopus y *Biblioteca Virtual de Saúde*, así como búsquedas manuales en las listas de referencias de los artículos seleccionados. El acceso a las bases de datos se realizó a través del Portal de Revistas de la *Coordenação de Aperfeiçoamento de Pessoal de Nível Superior* (CAPES), con base en la identificación a través de la Comunidad Académica Federada (CAFe), como forma de estandarizar la recopilación en estas bases de datos.

Las búsquedas se realizaron utilizando descriptores y/o términos alternativos, en idioma inglés, que aparecen en los Descriptores en Ciencias de la Salud (DeCS) y *Medical Subject Headings* (MeSH), como se muestra en la Figura 1.

**Figura 1-:** Estrategia de búsqueda en la base de datos. São Carlos, SP, Brasil, 2024

**Base eletrónica**	**Estrategia utilizada en la búsqueda**
*PubMed Central*	(Long COVID[Abstract] OR Post-Acute COVID-19 Syndrome[Abstract]) AND Pain[Abstract]
*Web of Science*	((AB=(“Long COVID”)) OR AB=(“Post-Acute COVID-19 Syndrome”)) AND AB=(Pain)
*Biblioteca Virtual em Saúde*	(ab:(long COVID)) OR (ab:(post-acute COVID-19 syndrome)) AND (ab:(pain)) AND instance:“lilacsplus”
Scopus	(ABS ( “Long COVID” ) OR ABS ( “Post-Acute COVID-19 Syndrome” ) AND ABS ( pain ) ) AND PUBYEAR > 2019 AND PUBYEAR < 2025 AND ( LIMIT-TO ( DOCTYPE, “ar” ) )

### Criterios de elegibilidad

Los criterios de inclusión fueron estudios primarios publicados en portugués, inglés y español entre marzo de 2020 y junio de 2024. Se excluyeron artículos duplicados, de opinión, editoriales, revisiones, información de sitios *web*, estudios cuyos títulos y resúmenes no encuadraban en el alcance de la investigación y artículos que no estaban disponibles en su totalidad y de forma gratuita.

### Procesamiento, análisis y extracción de datos

La búsqueda de artículos se realizó en las bases de datos electrónicas en julio y el proceso de selección de estudios tuvo lugar de agosto a octubre de 2024. Para ayudar al proceso de selección de estudios, luego de implementar la estrategia de búsqueda en cada base de datos, las referencias fueron importadas a la aplicación *StArt (State of the Art through Systematic Review*^(17)^, una herramienta de revisión desarrollada por el Laboratorio de Investigación en Ingeniería *de Software* (LaPES) de la *Universidade Federal de São Carlos* (UFSCar). La selección de los estudios se realizó en dos etapas: la primera consistió en la lectura de títulos y resúmenes, seguida de la lectura del artículo completo. Los estudios elegibles fueron recuperados en su totalidad y evaluados por dos investigadores. En ambas etapas se discutieron las discrepancias hasta llegar a un consenso, y posteriormente se procedió a la selección final.

La extracción de datos se realizó mediante una hoja de cálculo *de Microsoft Excel* que contiene campos para registrar información relevante de los estudios primarios, como la identificación del estudio (autores, año, país de publicación y objetivo), las características metodológicas (tipo de estudio y tamaño de la muestra) y los resultados clave relacionados con las características demográficas y clínicas, así como con el dolor como síntoma de COVID persistente (localización, frecuencia, duración, intensidad y calidad). Esta herramienta se diseñó para garantizar la uniformidad y facilitar la organización de los datos, además de permitir el posterior análisis cuantitativo y cualitativo de los resultados.

El proceso de selección de estudios se presenta en un diagrama de flujo y los resultados extraídos de las publicaciones se presentan en figuras (cuadros), en formato descriptivo.

La preparación de esta revisión también se adhirió a las recomendaciones del *Preferred Reporting Items for Systematic Reviews and Meta-analyses Extension for Scoping Reviews* (PRISMA-ScR)^(18)^.

### Aspectos éticos

En cuanto a los aspectos éticos de la investigación, no hubo discriminación en la selección de artículos o estudios, respetándose el criterio de cegamiento individual.

## Resultados

Se identificaron 1421 artículos en las bases de datos, de los cuales 809 se excluyeron por tratarse de material duplicado, 515 tras leer los títulos y resúmenes, y 78 tras leer el texto completo. Por lo tanto, se seleccionaron para el estudio 19 artículos que abordaban el dolor como síntoma de COVID persistente (Figura 2).

**Figura 2:** – Diagrama de flujo de referencias: inclusión y exclusión de artículos. São Carlos, SP, Brasil, 2024

Las 19 (100%) publicaciones incluidas en esta revisión de alcance se publicaron en inglés. En cuanto a los países participantes, 5 (26,3%) estudios se realizaron en Estados Unidos, 4 (21%) en Turquía, 3 (15,6%) en España y 1 (5,3%) en cada uno de los siguientes países: Irán, Indonesia, Chipre, Japón, Brasil, México y el Reino Unido.

En cuanto al tipo de estudios, 9 (47,3%) fueron estudios transversales, 5 (26,3%) estudios de cohorte, 4 (21,1%) estudios de caso y 1 (5,3%) estudio de casos y controles. La Figura 3 presenta la información relevante de cada artículo seleccionado, incluyendo autores, año de publicación, país de estudio, diseño y muestra del estudio, y resultados principales.

**Figura 3-:** Descripción de los artículos, según autor, año, ubicación, objetivo, tipo de estudio y características demográficas y clínicas. São Carlos, SP, Brasil, 2024

**N** *	**Autor, año y lugar**	**Objetivo**	**Tipo de estudio**	**Características demográficas y clínicas**
1	Arostegui, 2021, Estados Unidos ^( 19 )^	Describir un caso de síntomas gastrointestinales en un niño tres meses después del diagnóstico de COVID-19 ^†^ .	Estudio de caso.	N ^‡^ = 1, 11 años, sexo femenino. La niña desarrolló síntomas gastrointestinales durante la fase aguda de la infección por COVID-19 ^†^ y esos síntomas persistieron tres meses después. Tras tres semanas de tratamiento, el dolor abdominal no había remitido.
2	Savarraj, 2021, Estados Unidos ^( 20 )^	Caracterizar los resultados neurológicos a largo plazo después del COVID-19 ^†^ .	Estudio de cohorte.	N ^‡^ = 58, media de edad 49,2 años y 54% del sexo masculino. Se incluyeron personas con antecedentes de hospitalización por COVID-19 ^†^ . El síntoma más común en la muestra fue el dolor (60%). Quienes reportaron dolor eran significativamente más jóvenes (45 frente a 53 años; p ^§^ < 0,05). Las mujeres presentaron una mayor frecuencia de dolor; la diferencia no fue estadísticamente significativa, y no hubo variación en la etnia ni en los antecedentes médicos.
3	Azadvari, 2022, Irán ^( 21 )^	Evaluar la prevalencia de diferentes síntomas musculoesqueléticos y sus factores asociados en pacientes con COVID persistente ^†^ .	Estudio transversal.	N ^‡^ = 239, media de edad 37,96 años; 69,9% del sexo femenino. En la muestra, la mialgia (80,3%) y la cefalea (62,3%) fueron los síntomas más comunes. El índice de masa corporal y los días perdidos por COVID-19 ^†^ fueron significativamente mayores en los pacientes que experimentaron dolor torácico, dolor en las piernas, artralgia y mialgia durante períodos más prolongados (p ^§^ < 0,05). El sexo femenino se asoció con un mayor riesgo de encamarse por dolor (p ^§^ = 0,037).
4	Peñas, 2022, España ^( 22 )^	Identificar la correlación entre la magnitud del dolor y los síntomas sensoriales, asociados a variables de sensibilización, cognitivas y psicológicas en sobrevivientes de COVID-19 ^†^ con dolor post-COVID ^†^ .	Estudio transversal.	N ^‡^ = 146, media de edad 57,5 años; 54,11% del sexo femenino. Se incluyeron personas con antecedentes de hospitalización por COVID-19 ^†^ . A mayor extensión del dolor, menor intensidad (p ^§^ = 0,014). La asociación entre la intensidad y la extensión del dolor fue significativa, aunque débil, en hombres (p ^§^ = 0,006), pero no en mujeres con dolor post-COVID ^†^ .
5	Peñas, 2022, España ^( 23 )^	Investigar la asociación entre biomarcadores serológicos de gravedad del COVID-19 ^†^ al ingreso hospitalario con síntomas de dolor post-COVID ^†^ en sobrevivientes del COVID-19 ^†^ previamente hospitalizados.	Estudio de cohorte.	N ^‡^ = 412, media de edad 62 años; 53,9% del sexo masculino. La prevalencia del dolor fue del 42,7% y del 36,2% a los 6,8 y 13,2 meses tras el alta hospitalaria. Los pacientes que desarrollaron dolor post-COVID ^†^ 6 meses después del alta hospitalaria presentaron mayor número de síntomas al ingreso y mayor número de comorbilidades que quienes no experimentaron dolor post-COVID ^†^ a los 6 meses. Entre quienes desarrollaron dolor post-COVID ^†^ , el 51,7% ya presentaba dolor previo, con exacerbación del dolor en el 16,4% de los casos.
6	Güven, 2022, Turquía ^( 24 )^	Identificar predictores de COVID ^†^ prolongado mediante el análisis de los hallazgos clínicos y de laboratorio y las características demográficas de los niños con COVID-19 ^†^ .	Estudio de cohorte.	N ^‡^ = 500, media de edad 12,75 años (COVID ^†^ positivo) y 12,50 años (grupo control). En ambos grupos el 50,2% eran del sexo femenino. En general, el 12,4% de los niños reportaron síntomas de COVID persistente ^†^ . Los síntomas fueron significativamente más frecuentes en pacientes con signos de infección de las vías respiratorias superiores en la exploración física (p = 0,025).
7	Mutiawati, 2022, Indonesia ^( 25 )^	Determinar las características del dolor de cabeza en pacientes post-COVID-19 ^†^ en Indonesia.	Estudio transversal.	N ^‡^ = 215, 45,6% tenían entre 30 y 39 años; 69,8% eran del sexo femenino. El 72,1% de los participantes experimentó cefaleas durante la hospitalización o el autoaislamiento por COVID-19. Factores como la actividad física, el ruido y la luz intensa, o una combinación de ambos, agravaron las cefaleas, mientras que el descanso, los analgésicos y la combinación de ambos contribuían a aliviarlas.
8	McHarg, 2022, Estados Unidos ^( 26 )^	Caracterizar sistemáticamente los síntomas oculares encontrados en la mayoría de las personas no hospitalizadas con infección por COVID-19 ^†^ .	Estudio transversal.	N ^‡^ = 229, media de edad 42,5 años; 78,1% del sexo femenino. El 24,5% de la muestra reportó dolor ocular. Los síntomas oculares fueron más frecuentes en personas con antecedentes oculares; solo el 13,3% acudió a oftalmología, y de estos, el 40,9% atribuyó sus síntomas al COVID- 19 ^†^ .
9	Topal, 2022, Turquía ^( 27 )^	Investigar el dolor musculoesquelético prolongado como síntoma de la condición post-COVID-19 ^†.^	Estudio transversal.	N ^‡^ = 501, media de edad 39 años y 51,3% eran del sexo masculino. Sesenta y nueve participantes reportaron dolor prolongado Post-COVID ^†.^ El 58% eran del sexo femenino, sin diferencias significativas entre géneros. La incidencia de comorbilidad general o la presencia de una o más afecciones adicionales concomitantes con el COVID-19 ^†^ fue mayor en pacientes con dolor prolongado.
10	Zha, 2022, Estados Unidos ^( 28 )^	Describir un informe de caso de dolor miofascial después de la infección por COVID-19 ^†^_._	Estudio de caso.	N ^‡^ = 1, 59 años, sexo masculino El paciente refirió incapacidad para realizar actividades de la vida diaria debido a disnea de esfuerzo persistente, confusión mental y mialgia difusa. Después de 12 meses, los síntomas empeoraron, asociados con un aumento significativo del estrés psicosocial, lo que contribuyó a su exacerbación.
11	Zis, 2022, Chipre ^( 29 )^	Estimar la prevalencia del dolor crónico en general y del dolor neuropático en particular, describir los síndromes de dolor más comunes e identificar los determinantes del dolor en una población aleatoria de sobrevivientes de COVID-19 ^†^ .	Estudio transversal.	N ^‡^ = 90, media de edad 47,5 años; 62,2%. El 63,3% de los participantes presentaba dolor crónico, más frecuente en personas mayores (50 vs. 42 años) y mujeres. Tras ajustar por edad y sexo, la cefalea durante el COVID-19 ^†^ fue un predictor significativo de dolor neuropático, con un riesgo 4,9 veces mayor.
12	Mateo, 2023, España ^( 30 )^	Evaluar la calidad de vida relacionada con la salud y las características del dolor en pacientes con COVID ^†^ prolongado y comparar la ubicación del dolor con aquellos que se recuperaron exitosamente de COVID-19 ^†^ .	Estudio de casos y controles.	N ^‡^ = 213, en el grupo COVID prolongado ^†^ , media de edad 44,99 años; 75,26% del sexo femenino. La prevalencia, intensidad y propagación del dolor fueron significativamente mayores en personas con COVID ^†^ persistente en comparación con las personas recuperadas y controles sanos.
13	Erden, 2023, Turquía ^( 31 )^	Investigar los síntomas musculoesqueléticos, el tipo de dolor y el efecto sobre la calidad de vida en pacientes que experimentan dolor después de COVID-19 ^†^ .	Estudio transversal.	N ^‡^ = 97, media de edad 46,5 años; 69,1% del sexo masculino. La puntuación media de la escala visual analógica en mujeres, así como el número de pacientes que desarrollaron mialgia tras el COVID-19 ^†^ , fueron significativamente mayores que en los hombres (p§ <0,05). En las personas con dolor musculoesquelético previo, el dolor se agravó tras la infección por COVID-19 ^†^ .
14	Fujita, 2023, Japón ^( 32 )^	Explicitar las características de los dolores de cabeza en pacientes con COVID ^†^ prolongado, centrándose en la historia y las condiciones clínicas de los pacientes y comprender el impacto de los dolores de cabeza en la calidad de vida de estos pacientes.	Estudio de cohorte.	N ^‡^ = 482, media de edad 37 años; 56% del sexo femenino. El 23,4% de los participantes presentó cefalea. En la fase aguda del COVID-19 ^†^ , la mayoría de los casos de COVID ^†^ persistente fueron leves, tanto en el grupo con cefalea (88,5%) como en el grupo sin cefalea (82,7%).
15	Rodrigues, 2023, Brasil ^( 33 )^	Evaluar las características clínicas de la cefalea por COVID ^†^ persistente.	Estudio transversal.	N ^‡^ = 102, 62,7% tenían más de 39 años; 81,37% eran mujeres. La cefalea en el COVID ^†^ prolongado se observó con mayor frecuencia en mujeres mayores de 39 años. El 49,0 % de los participantes tenía antecedentes de cefalea y reportó empeoramiento del dolor durante la etapa de COVID ^†^ prolongado.
16	Yadav, 2023, Estados Unidos ^( 34 )^	Descripción de informes de casos de cinco pacientes que desarrollaron dolor articular varias semanas después de la recuperación de la infección aguda por COVID-19 ^†^ .	Estudio de caso.	N ^‡^ = 5, mujeres, edad entre 19 y 61 años. la infección por COVID-19 ^†^ ; por lo tanto, no se consideraron COVID ^†^ prolongado. El primer caso involucró a una mujer de 45 años con obesidad mórbida y osteoartritis, quien experimentó una mejoría del 70-80% en los síntomas después de tres meses de terapia con corticosteroides. El segundo caso involucró a una mujer de 46 años sin comorbilidades que persistió con dolor durante siete meses, sin respuesta significativa al tratamiento reumatológico.
17	Salazar, 2024, México ^( 35 )^	Describir un informe de caso de síntomas de mialgia y artralgia después de la infección por COVID-19 ^†^ .	Estudio de caso.	N ^‡^ = 1, mujer, 75 años. El paciente no tenía antecedentes de enfermedades autoinmunes ni reumáticas. Tras el tratamiento farmacológico, se recuperó completamente tras 6 meses de seguimiento.
18	Khoja, 2024, Reino Unido ^( 36 )^	Investigar las características clínicas del dolor crónico reciente en pacientes con COVID ^†^ prolongado y su impacto en la función física, el estado de ánimo y la calidad de vida.	Estudio transversal.	N ^‡^ = 30, 63% eran del sexo femenino y la edad media fue 46,8 años. El dolor se asoció con sensibilización central, citocinas proinflamatorias elevadas, debilidad y reducción de la función y la actividad física.
19	Akarsu, 2024, Turquía ^( 37 )^	Determinar la frecuencia y características del dolor persistente en individuos diagnosticados con COVID-19 ^†^ y compararlos con aquellos que se recuperaron o no experimentaron dolor persistente.	Estudio de cohorte.	N ^‡^ = 191, 51,8% eran del sexo masculino, edad media 41,4 años. Predominio significativo de cefaleas en pacientes mujeres (p ^§^ < 0,001). Treinta y nueve pacientes reportaron una exacerbación de la cefalea previa a la infección. En comparación con otros pacientes con COVID-19 ^†^ , quienes presentaron características de dolor neuropático persistente fueron mujeres (p ^§^ = 0,02), mayores (p ^§^ = 0,02) y presentaron una mayor incidencia de síntomas como dolor de garganta (p ^§^ = 0,003) y cefalea (p ^§^ = 0,04).

*N = Número; ^†^COVID = *Coronavirus Disease*; ^‡^N = Muestra del estudio; ^§^p = Nivel de significancia

Entre los 19 estudios analizados, se observó que el COVID prolongado se manifiesta en todas las edades, desde niños hasta la población de edad avanzada^(19-37)^. Al analizar la relación entre el grupo de edad y el desarrollo de dolor en el COVID prolongado, un estudio mostró que los adultos que reportaron síntomas de dolor en el COVID prolongado eran más jóvenes que aquellos que no reportaron dolor (45 vs. 53 años)^(20)^; sin embargo, se observó lo contrario en otro estudio, en el que los adultos que desarrollaron dolor crónico, en comparación con los individuos sin dolor, eran mayores (50 vs. 42 años)^(29)^.

En cuanto al sexo, en diez de los estudios analizados, la muestra estuvo compuesta predominantemente por mujeres^(19,22,24-26,29-30,33-34,36)^. En lo que se refiere al análisis del sexo y la aparición del dolor en el COVID persistente, los estudios indicaron que las mujeres eran más propensas al dolor crónico^(29,37)^, además de presentar niveles más altos de intensidad del dolor^(22,31)^.

En lo atinente a la presencia de comorbilidades, cabe destacar que un estudio no encontró asociación entre la presencia de comorbilidades y personas con o sin dolor^(20)^; por otro lado, otro estudio mostró que un índice de masa corporal (IMC) más alto se asoció con periodos más prolongados de manifestación de síntomas musculoesqueléticos, como dolor torácico, artralgia y mialgia^(21)^. Además, en otro estudio, se identificó que la presencia de comorbilidades fue mayor en personas con dolor prolongado^(27)^.

Los estudios también han demostrado que, incluso después de iniciar algún tratamiento para los síntomas persistentes de COVID-19, algunas personas persistieron con síntomas o estos no se eliminaron por completo^(19,34)^. Además, estudios han demostrado que las personas con dolor antes de la infección por COVID-19 experimentaron una exacerbación del dolor después de la infección^(23,31,33,37)^.

En la descripción de la localización del dolor, como se muestra en la Figura 4, las regiones más frecuentes mencionadas en los estudios fueron: cabeza^(20,24-25,28,30,32-33,37)^; región torácica^(19-23,27-28,34)^; cuello^(22,27-31,34-35)^; espalda^(20,22-23,27-28,31,37)^; hombro^(22-23,28,31,34-36)^; zona lumbar^(21,24,27,29,31,36)^; cadera^(21-22,31,34)^; manos^(23,29,31,34)^ ; rodillas^(22-23,31,34,36)^; piernas^(28,30,34)^; articulaciones^(22,29,31,35-36)^; muslo^(29,31)^; brazos^(28,31)^; pies^(29,31)^; abdomen^(19,24)^ y solo un estudio abordó el dolor ocular^(26)^.

**Figura 4-:** Descripción del dolor según localización, frecuencia, duración, intensidad y calidad. São Carlos, SP, Brasil, 2024

**N** *	**Ubicación**	**Frecuencia y duración**	**Intensidad**	**Calidad**
1	Abdominal (cuadrante superior e inferior derecho)	Frecuencia: intermitente Duración: mayor a tres meses	No medido	Cólicos o ardor
2	Espalda (17,0%), pecho (14,2%), cabeza (14,2%)	Frecuencia: no descripta Duración: no descripta	Escala utilizada: *Pain, Enjoyment, General Activity* (0-10) Espalda: puntuación media 6,3 (moderado) Pecho: puntuación media 3 (leve) Cabeza: puntuación media 7,6 (intenso)	No descripto
3	Cabeza, cuello, espalda, zona lumbar, caderas, piernas, pecho	Frecuencia: no descripta Duración: no descripta	No medido	No descripto
4	Rodillas (18%), cabeza (15%), cuello (13%), hombros (13%), espalda (9%)	Frecuencia: no descripta Duración: media de 18,8 meses	Escala utilizada: Calificación numérica del dolor (0-10) Mujeres: puntuación media 5,9 (moderado) Hombres: puntuación media 5,2 (moderado)	No descripto
5	Generalizado (22,7%), tórax/pecho (19,9%), extremidad inferior (11,5%), columna cervical (8,5%), hombros (8,5%), columna lumbar (7,9%), extremidad superior (6,8%), muñeca/codo (5,7%), rodilla (5,7%), cadera (2,8%)	Frecuencia: no descripta Duración: mayor a 6 meses	No medido	No descripto
6	Articulaciones (7,6%), zona lumbar (4,8%), cabeza (3,2%), abdominales (2,0%)	Frecuencia: no descripta Duración: media 5,32 meses	No medido	No descripto
7	Cabeza (72,1%): difusa 47,4%, frontoparietal 5,6%, frontal 5,1% y parietal 4,2%	Frecuencia: 1–2 veces al mes (38,6%), 1–2 veces/semana (14,9%), más de 2 veces/semana (7,4%), diariamente (39,1%) Duración: no descripta	Escala utilizada: Calificación numérica del dolor (0-10) Moderado (46,5%), leve (42,3%) e intenso (11,1%)	Pulsátil (59,1%), presión (20,9%), mixto (6,0%), punzante (5,1%), ardor (1,9%)
8	Ocular (24,5%): bilateral 90,9%	Frecuencia: no descripta Duración: más de 14 días	No medido	No descripto
9	Cuello, brazo, espalda, cintura.	Frecuencia: no descripta Duración: media 4,38 meses	Escala utilizada: Numérica visual analógica del dolor (0-10) Puntuación media 7,2 (intenso)	Ardor (23,2%), entumecimiento (21,7%), hormigueo (14,5%), escozor (5,8%), congelación (1,4%)
10	Cuello, hombros, parte superior de la espalda, parte superior posterior bilateral de los brazos y parte inferior posterior de las piernas.	Frecuencia: no descripta Duración: 12 meses	Escala utilizada: Escala Likert del dolor (0-10) Puntuación 6 (moderado)	No descripto
11	Lumbar (37,8%), articulaciones (28,9%), cuello (12,2%)	Frecuencia: no descripta Duración: no descripta	Escala utilizada: *Brief Pain Inventory* (0-10) Puntuación media 5,12 (moderado)	No descripto
12	Cuello (69,1%), piernas (68%), cabeza (63,9%)	Frecuencia: no descripta Duración: promedio 104 semanas	Escala utilizada: *Brief Pain Inventory* (0-10) Puntuación media 5,12 (moderada)	No descripto
13	Artralgia: hombro (44,3%), rodilla (40,2%), codo (26,8%), cadera (22,7%), muñeca (21,9%), pie/tobillo (13,4%) Mialgia: cuello/espalda/lumbar (60,8%), pantorrilla (45,4%), brazo (44,3%), antebrazo (38,1%), muslo (15,5%), parte delantera del tronco (16,5%)	Frecuencia: no descripta Duración: promedio 4,0 meses	Escala utilizada: Numérica visual analógica del dolor (0-10) Puntuación general: 7 (intensa) Puntuación femenina: media 7 (intenso) Puntuación masculina: media 6 (moderado)	No descripto
14	Cabeza (23,4%)	Frecuencia: no descripta Duración: no descripta	No medido	No descripto
15	Cabeza (occipital 23,3%, bitemporal 22,8%, frontal 21,2%, difusa 18,9%, unilateral 14,1%)	Frecuencia: diaria (27,5%), 2–5x/semana (44,1%), semanal (18,6%), mensual (9,8%) Duración: media 321,46 días	Escala utilizada: Numérica visual analógica del dolor (0-10) Puntuación 5 (65,7% - moderado) y 8 (25,5% - intenso)	Presionante (57,8%), pulsante (27,5%), ardiente (6,9%), mixto (7,8%)
16	Caso 1: Rodillas, hombros, manos, espalda, pecho, cuello, mandíbula, muñeca, rodilla y tobillo. Caso 2: rodillas, hombros, tobillos, caderas, lumbar, cuello y muñecas	Frecuencia: no descripta Duración: Mayor de 3 meses (caso 1) Mayor de 7 meses (caso 2)	No medido	No descripto
17	Cuello, hombros, antebrazos	Frecuencia: no descripta Duración: promedio 6 meses	Escala utilizada: no se describe en el estudio Calificación: intenso	No descripto
18	Rodillas (70%), hombros (63%), columna cervical (60%) y región lumbosacra (57%)	Frecuencia: intermitente (episódica) (10%), continuo (90%) Duración: media 519,1 días	Escala utilizada: *Brief Pain Inventory* (0-10) Puntuación media 5,3 (moderado)	Dolor sordo y persistente 28 (93%) Afilado y penetrante 2 (7%)
19	Cabeza (29,8%): bilateral 80,7%) Mialgia: extremidades (54,5%), espalda (36,4%), generalizada (9,1%)	Frecuencia Cabeza: Menos de 1 día/mes (5,3%) 1–4 días/mes (73,7%) 5–14 días/mes (12,3%) Los 15 días más grandes (8,8%) Frecuencia de otras localizaciones: no descripta. Duración: 1,5 años	Escala utilizada: clasificación según los autores Cabeza: Leve (29,8%) Moderado (33,3%) Intenso (36,9%) Escala utilizada: no se describe en el estudio Mialgia: Puntuación media 6,3	Cabeza: Pulsante (47,4%) presionante (38,6%) ardor (3,5%) puntada (5,3%)

*N = Número

En cuanto a la frecuencia, puede presentarse diariamente, así como en episodios mensuales^(19,25,33-34,36-37)^; en cuanto a la duración del dolor en COVID prolongado, algunos estudios indicaron que la manifestación del dolor permaneció durante algunos meses; otros análisis mostraron la persistencia del síntoma durante más de un año ^(19,22-24,26-28,30-31,33-37)^.

Respecto a los estudios que abordaron las características de la calidad del dolor, los descriptores reportados fueron cólico, ardor, pulsátil, presión, punzante, quemante, entumecimiento, congelamiento, penetrante o mixto^(19,25,27,33,36,37)^. Respecto a las características de la cefalea, dos estudios demostraron que puede ser aguda, punzante, opresiva, urente, pulsátil o mixta; además, puede afectar toda la cabeza o una región específica^(25,33,37)^. Para evaluar la intensidad del dolor, los estudios utilizaron diferentes escalas, y se observó que el dolor variaba de leve a intenso en las muestras^(20,22,25,27-31,33-37)^.

## Discusión

El análisis de los estudios incluidos en esta revisión reveló que el dolor como síntoma de COVID persistente puede afectar a todas las edades, siendo más prevalente entre las mujeres. El dolor que se manifiesta en el COVID persistente puede afectar áreas de todo el cuerpo; sin embargo, las localizaciones más comunes descriptas en los estudios analizados fueron el tórax, la cabeza, el cuello, los hombros, la zona lumbar y la cadera. En cuanto a la calidad del dolor, este puede presentarse de diferentes maneras según la localización, como cólicos, ardor, pulsátil, presión, punzadas, escozor, entumecimiento, congelamiento, puntadas o mixto. La intensidad del dolor varió de leve a intenso, con frecuencia diaria a intermitente, y la duración varió de unos pocos meses a años. Este análisis también mostró que las personas que experimentaban dolor antes de la infección por COVID-19 pueden experimentar una exacerbación después de la infección viral.

La fisiopatología del dolor en el COVID-19 persistente sigue siendo incierta; sin embargo, entre las posibles explicaciones, destaca la diversidad de síntomas que se manifiestan en la enfermedad debido a la naturaleza multisistémica de la infección por SARS-CoV-2 en la fase aguda, que involucra varios órganos y sistemas. Por lo tanto, la región donde se manifiesta el dolor podría estar relacionada con secuelas multiorgánicas, es decir, el sitio de daño tisular debido a la invasión viral en la fase aguda de la infección. Este daño puede considerarse el desencadenante inicial de los síntomas^(38-39)^.

Sin embargo, la persistencia o el desarrollo de síntomas después de la fase aguda de la infección, que caracteriza al COVID prolongado, puede estar relacionada con mecanismos distintos al daño tisular, como la persistencia de virus residual en el organismo. En este escenario, un estudio demostró la persistencia del SARS-CoV-2 en el intestino delgado cuatro meses después del inicio del COVID-19 en la mitad de la muestra analizada^(40)^. Así, además del tracto respiratorio, los órganos que expresan el receptor de la enzima convertidora de angiotensina 2 (ECA2), incluidos los fluidos corporales, la saliva, la orina, la sangre, las lágrimas y el semen, pueden actuar como reservorio viral, y la persistencia del virus puede desencadenar mecanismos inflamatorios crónicos^(38,41)^.

Por lo tanto, los mecanismos inflamatorios a largo plazo tras una infección viral, resultantes de una respuesta inmunitaria desregulada, además de los factores de riesgo asociados del individuo infectado, como la presencia de enfermedades preexistentes, pueden contribuir a la pérdida de autotolerancia y la hiperinflamación, así como a la hiperactivación de procesos tromboembólicos/coagulantes, multiorgánicos y del sistema nervioso autónomo. Así, la disfunción presente en el organismo puede mantener o perpetuar los procesos patológicos, lo que lleva a la cronicidad de estos síntomas en los órganos y sistemas afectados^(38-39)^.

Además del desarrollo del dolor como síntoma de COVID persistente, la literatura sugiere que las personas con dolor previo a la infección por SARS-CoV-2 pueden experimentar una exacerbación después de la infección viral. En este escenario, una hipótesis es que los mecanismos inflamatorios del virus podrían contribuir a la hiperexcitabilidad de los sistemas nerviosos periférico y central a través de diferentes vías, lo que favorece la aparición de dolor Post-COVID o su empeoramiento^(23,42)^.

En la literatura se han identificado algunos factores que aumentan el riesgo de desarrollar síntomas como el dolor asociado con el COVID-19 persistente, como la necesidad de hospitalización por COVID-19, el género, la edad y las comorbilidades. En cuanto a la hospitalización, los estudios seleccionados no presentaron comparaciones en las manifestaciones de dolor entre personas hospitalizadas y no hospitalizadas por COVID-19.

Por otro lado, análisis en España y Brasil mostraron que la persistencia de síntomas prolongados de COVID fue más prevalente en el grupo hospitalizado que en el grupo de personas sin necesidad de hospitalización, siendo la mialgia reportada con mayor frecuencia en el grupo hospitalizado^(43-44)^.

Sin embargo, es importante considerar que las personas que requirieron hospitalización en cuidados intensivos pueden experimentar algún tipo de síndrome post-cuidados intensivos, que generalmente ocurre después de una enfermedad crítica prolongada. Además, los sobrevivientes pueden experimentar inflamación persistente, inmunosupresión y disfunción orgánica crónica durante la hospitalización^(38-39,45)^. Por lo tanto, la literatura ha descripto que los síntomas de COVID persistente también pueden estar asociados con el síndrome de cuidados intensivos, así como una combinación del síndrome resultante de la hospitalización más las secuelas multiorgánicas y la disfunción inmunitaria resultante de la infección por SARS-CoV-2^(38)^.

Otro factor de riesgo frecuentemente destacado en la literatura en los síntomas generales del COVID prolongado, pero especialmente en la manifestación del dolor, es el sexo femenino, asociado a mayor duración de los síntomas y limitaciones funcionales en comparación con los individuos del sexo masculino^(44-46)^.

Aunque pocos estudios seleccionados para esta revisión mostraron una asociación entre el género femenino y el desarrollo de COVID persistente, se observó que las mujeres representaban la mayoría de las muestras. Un estudio realizado en el Reino Unido reveló que las mujeres tenían un mayor riesgo de desarrollar síntomas generales de COVID persistente en comparación con los hombres^(47)^. Otro estudio de revisión mostró que las mujeres, en comparación con los hombres, tenían mayor probabilidad de desarrollar mialgia en el COVID persistente^(46)^.

En este escenario, la literatura apunta a la existencia de dimorfismo sexual en la mayoría de las condiciones de dolor crónico, incluyendo artritis, migraña y fibromialgia, con una prevalencia mayor en mujeres que en hombres^(48)^. Entre las hipótesis para el dimorfismo sexual en el dolor, se describe la influencia de las hormonas sexuales en la sensibilidad al dolor. Así, se sugiere que la testosterona es antinociceptiva y protectora, es decir, disminuye la sensibilidad al dolor, mientras que el estradiol y la progesterona tienen efectos pro y antinociceptivos. Por lo tanto, las mujeres con altos niveles de estradiol tienen una sensibilidad al dolor disminuida y una efectividad analgésica aumentada en comparación con las mujeres con niveles bajos de estradiol. Además, la sensibilidad al dolor de las mujeres cambia a lo largo del ciclo menstrual, con una mayor sensibilidad durante la fase lútea, lo que indica una fuerte influencia de las hormonas sexuales en el dolor^(49)^.

Asimismo, en cuanto a los factores de riesgo para el desarrollo de COVID persistente, los estudios seleccionados para esta revisión no identificaron enfoques sobre la raza y el dolor en el COVID prolongado. Sin embargo, especialmente en el dolor crónico, se señala que los grupos racialmente marginados sufren de forma desproporcionada síntomas que no se alivian como resultado de percepciones erróneas sobre la intensidad del dolor durante la interacción médico-paciente y del acceso desigual a la información y la atención médica^(50)^.

Los análisis de esta revisión muestran que el COVID-19 prolongado puede afectar a personas de todas las edades, desde niños hasta ancianos^(19-34)^. Sin embargo, aunque la edad avanzada puede no parecer un factor determinante en el desarrollo del COVID-19 prolongado, existe evidencia de que el dolor puede presentarse de forma heterogénea según el país de residencia. En este contexto, un análisis de más de 100.000 participantes de 20 países mostró que, en personas de 50 a 80 años en países como Corea, Israel y Eslovenia, la intensidad del dolor reportado aumentó a medida que aumentaba la edad. Sin embargo, en países como Estados Unidos y Dinamarca, la asociación entre la intensidad del dolor y el aumento de la edad fue insignificante, con poca variabilidad en la intensidad^(51)^.

En consecuencia, la intensidad del dolor no solo estaría relacionada con la edad cronológica, sino también con las condiciones en las que las personas envejecen. En algunos países de altos ingresos, las personas reciben información constante sobre las posibles opciones de tratamiento del dolor y, por lo tanto, enfrentan menos barreras para acceder a los servicios de evaluación y tratamiento^(51)^. Por lo tanto, al evaluar el dolor, se destaca la importancia de incluir, además de los aspectos biológicos, las condiciones socioeconómicas a las que las personas están expuestas, ya que estas pueden influir directamente en la percepción de la intensidad del dolor.

Otro punto de divergencia encontrado en los estudios seleccionados para esta revisión se refiere a la asociación de comorbilidades como factor de riesgo para la manifestación del dolor como síntoma de COVID prolongada. Un estudio en el Reino Unido informó que un IMC basal en el rango de sobrepeso u obesidad se asoció con un mayor riesgo de síntomas persistentes, con aquellos con un IMC mayor de 30 kg/m2 en comparación con aquellos con un IMC de 18,5–25 kg/m2^(47)^. Otro análisis en Dinamarca mostró una mayor prevalencia de dolor entre pacientes con COVID prolongado con comorbilidades^(52)^. La asociación entre la presencia de comorbilidades y síntomas de COVID persistente puede atribuirse a la liberación excesiva de citocinas en la infección aguda por SARS-CoV-2, que puede agravar los mecanismos inflamatorios^(53)^.

Por lo tanto, si bien todas las personas que han tenido COVID-19 son susceptibles a desarrollar COVID persistente, algunas pueden tener un mayor riesgo de padecer la enfermedad. Cabe destacar que el dolor afecta la salud física y mental de las personas, además de impactar en su calidad de vida. Un estudio en Bangladesh mostró una relación inversa significativa entre el estado de salud física, la calidad de vida y la duración de los síntomas de COVID persistente^(54)^.

En ese marco, las personas con dolor, además del sufrimiento físico, pueden experimentar síntomas psicosociales e incluso impactos económicos, debido a la falta de comprensión por parte de la familia y los profesionales de la salud, e incluso la pérdida del empleo debido a los síntomas. Además, dado que el tratamiento del dolor crónico suele ser a largo plazo, el costo financiero de su manejo puede constituir una barrera, ya que requiere una gestión interdisciplinaria y los servicios de salud a menudo no brindan toda la atención necesaria ni la brindan de manera oportuna. Las dificultades financieras para financiar el tratamiento pueden resultar en una baja adherencia o abandono del tratamiento^(55)^.

En este contexto, es importante destacar que la vacunación contra el COVID-19 se ha sugerido firmemente como un factor protector contra el COVID persistente^(45)^. Un metaanálisis mostró que las personas vacunadas tenían un menor riesgo de desarrollar cualquier síntoma de COVID persistente en comparación con las personas no vacunadas. En comparación con los pacientes no vacunados, la vacunación mostró un efecto protector en pacientes vacunados con dos dosis, pero no con una dosis. Además, la vacunación fue eficaz contra el COVID persistente en pacientes vacunados antes de la infección por SARS-CoV-2 o vacunados después de la infección por SARS-CoV-2^(56)^.

La literatura muestra que, incluso después de iniciar algún tratamiento para el dolor en el COVID prolongado, algunas personas continúan con síntomas o estos no desaparecen por completo. Por lo tanto, se enfatiza que la adherencia a la vacunación es la mejor alternativa para reducir la probabilidad de desarrollar síntomas graves de la enfermedad, así como los síntomas de COVID persistente^(19,34,49)^.

También es importante destacar que cualquier enfoque de tratamiento clínico debe considerar las características específicas e individuales del dolor. Por ende, la exploración física para la evaluación del dolor debe abordar información sobre la localización, frecuencia, duración, intensidad y calidad^(57)^. En este análisis, se observó que la gran mayoría de los estudios se centran en la localización e intensidad del dolor, siendo la frecuencia, la duración y la calidad variables menos exploradas.

Cabe destacar que la frecuencia ayuda a identificar la persistencia o recurrencia del dolor, mientras que la calidad, incluyendo descripciones como pulsátil, quemante o punzante, entre otras, puede contribuir a comprender la naturaleza del dolor y desempeña un papel fundamental en la diferenciación y el diagnóstico de otras afecciones clínicas dolorosas^(58-59)^. Por lo tanto, la falta de patrones y características detalladas en el enfoque puede limitar una comprensión completa de la experiencia del dolor y su impacto en la calidad de vida de las personas afectadas por esta afección.

Además de una investigación detallada de las características del dolor reportado, la estandarización de la evaluación mediante instrumentos validados ayuda a monitorear la progresión clínica y optimizar la atención^(6,11,19-20)^. Para facilitar la evaluación adecuada del dolor en diferentes grupos de edad, existen instrumentos como la escala numérica, donde 0 corresponde a la clasificación “sin dolor” y 10 a “dolor máximo”. La escala facial, que puede utilizarse en personas con dificultades del habla o en niños, permite clasificar la intensidad del dolor según la expresión facial dibujada, con una expresión de felicidad que corresponde a la clasificación “sin dolor” y una expresión de máxima tristeza a la clasificación “dolor máximo”^(57)^.

Cabe destacar que el dolor resultante del COVID prolongado que persiste durante más de tres meses se califica como dolor crónico potencial. Puede considerarse una enfermedad invisible y, a menudo, los profesionales de la salud la consideran menos importante en comparación con otras quejas de los pacientes, minimizando así sus síntomas. Por lo tanto, recibir un diagnóstico formal de dolor no solo legitima la experiencia subjetiva del paciente, sino que también brinda la oportunidad de un manejo adecuado de los síntomas^(50)^. En este escenario, dada la incidencia del dolor en personas con COVID prolongada y el impacto biopsicosocial y económico que el dolor crónico puede causar, es esencial que los profesionales de la salud reconozcan las quejas de dolor.

Actualmente, la atención a las personas con COVID persistente está fragmentada; sin embargo, se recomienda que la asistencia se organice de manera integrada^(60)^. La identificación temprana de la enfermedad, el seguimiento continuo, la educación sanitaria y el fortalecimiento del autocuidado son esenciales para prevenir complicaciones y garantizar la derivación adecuada cuando sea necesario. En consecuencia, la integración entre los diferentes niveles de atención médica es esencial para el manejo eficaz del dolor, garantizando así la continuidad de la atención a las personas con COVID persistente^(59-62)^.

Hasta la fecha, no existen directrices consolidadas específicas para el manejo del dolor en el COVID-19 persistente. Sin embargo, las estrategias adoptadas incluyen intervenciones farmacológicas y no farmacológicas tradicionales, como, por ejemplo, fisioterapia, ejercicio gradual, terapia ocupacional y apoyo psicológico. Por lo tanto, la integración entre equipos multidisciplinares es esencial, ya que un manejo eficaz del dolor en el COVID-19 persistente requiere no solo el control sintomático, sino también la prevención de la cronicidad y la recuperación funcional^(12-14,61-62)^.

En este escenario, enfermería desempeña un papel estratégico en la atención a personas con dolor persistente en COVID prolongado, actuando tanto en la evaluación continua de los síntomas como en la implementación de intervenciones dirigidas al alivio del dolor. La aplicación sistemática de escalas validadas de medición del dolor, combinada con la monitorización de la respuesta a las terapias establecidas, permite ajustes tempranos en el plan terapéutico. Además, la enfermería desempeña un papel central en la educación para la salud, brindando orientación sobre autocuidado, adherencia a las terapias, prevención de exacerbaciones y manejo de factores desencadenantes. La escucha cualificada y el apoyo emocional brindados por el equipo de enfermería contribuyen a reducir el impacto psicosocial del dolor, promueven la rehabilitación y mejoran la calidad de vida^(12-14,62)^.

Las limitaciones de este estudio incluyen la inclusión de publicaciones exclusivamente en portugués, inglés y español, que pueden tener acceso restringido a evidencia relevante en otros idiomas, y la elección restringida de las bases de datos consultadas, ya que diferentes bases de datos presentan variaciones en la cobertura e indexación de revistas y tipos de publicaciones.

Sin embargo, los resultados de este análisis presentan implicaciones relevantes para el avance del conocimiento científico, en los campos de la salud y la enfermería. Comprender la diversidad en la localización y las características del dolor en el COVID persistente, que se manifiesta de diversas maneras, refuerza la complejidad de este síntoma y la necesidad de desarrollar protocolos de atención más eficaces e individualizados en la práctica de enfermería.

## Conclusión

Los resultados de esta revisión de alcance sugieren que el dolor en el COVID persistente puede afectar a personas de todas las edades, siendo más frecuente en mujeres. El dolor, como síntoma del COVID persistente, puede presentarse en cualquier zona del cuerpo; sin embargo, en algunas zonas, como la cabeza, el pecho, el cuello, los hombros, la zona lumbar y la cadera, se observan con mayor frecuencia. La intensidad puede variar de leve a grave, con una frecuencia diaria o intermitente, y la duración puede variar desde unos pocos meses hasta años, lo que sugiere una posible cronicidad de los síntomas.

Luego, los hallazgos de este estudio sobre las características clínicas y los patrones de dolor podrían contribuir al desarrollo de pautas específicas para el manejo de esta afección en personas con COVID persistente, lo que permitiría diferenciarlo de otras afecciones dolorosas. También destaca la necesidad de medidas preventivas, fomentando la adherencia a la vacunación contra el COVID-19 para reducir la probabilidad de desarrollar dolor como síntoma de COVID persistente, así como para reducir los costos financieros de los sistemas de salud al proporcionar un diagnóstico más preciso de la enfermedad.

Finalmente, a partir de esta revisión, se advierten importantes deficiencias en el enfoque de la evaluación del dolor en el COVID-19 persistente. La significativa falta de inclusión de las características del dolor, con pocos estudios que exploren en detalle aspectos como la frecuencia, la duración y la calidad del dolor en el COVID-19 persistente, podría contribuir a cambios en la forma en que se evalúa el dolor en la investigación y la práctica clínica, lo que ayudaría a orientar la investigación futura para mejorar la evaluación global del dolor.

Se sugiere que estudios futuros incluyan el análisis de variables individuales que puedan influenciar en la percepción del dolor como síntoma de COVID prolongado por parte de los profesionales de la salud, como la gravedad de la infección aguda por COVID-19, exámenes clínicos para evaluar indicadores inflamatorios, presencia de comorbilidades, tipo de ocupación, condiciones socioeconómicas y ambientales de la región de residencia y estado de vacunación contra el COVID-19, variables que pueden influir en las condiciones de salud y el desarrollo del dolor a largo plazo.

## Data Availability

Todos los datos generados o analizados durante este estudio están incluidos en este artículo publicado.
